# The impact of cardiovascular health and frailty on mortality for males and females across the life course

**DOI:** 10.1186/s12916-022-02593-w

**Published:** 2022-11-11

**Authors:** Jack Quach, Olga Theou, Judith Godin, Kenneth Rockwood, Dustin Scott Kehler

**Affiliations:** 1 School of Physiotherapy, Dalhousie University, Halifax, NS Canada; 2 Geriatric Medicine, Dalhousie University and Nova Scotia Health, NS Halifax, Canada

**Keywords:** Frailty, Cardiovascular Health, Mortality

## Abstract

**Background:**

The effect of frailty and poor cardiovascular health on mortality for males and females is not fully elucidated. We investigated whether the combined burden of frailty and poor cardiovascular health is associated with all-cause and cardiovascular disease (CVD) mortality by sex and age.

**Methods:**

We analyzed data of 35,207 non-institutionalized US residents aged 20–85 years old (mean age [standard deviation]: 46.6 [16.7 years], 51.4% female, 70.8% White, 10.3% Black, 13.2% Hispanic) from the National Health and Nutrition Examination Survey (1999–2015). Cardiovascular health was measured with the American Heart Association’s Life’s Simple 7 score (LS7). A 33-item frailty index (FI) was constructed to exclude cardiovascular health deficits. We grouped the FI into 0.1 increments (non-frail: FI < 0.10, very mildly frail: 0.1 ≤ FI < 0.20, mildly frail: 0.20 ≤ FI < 0.30, and moderately/severely frail: FI ≥ 0.30) and LS7 into tertiles (T1[poor] = 0–7, T2[intermediate] = 8-9, T3[ideal] = 10–14). All-cause and CVD mortality data were analyzed up to 16 years. All regression models were stratified by sex.

**Results:**

The average FI was 0.09 (SD 0.10); 29.6% were at least very mildly frail, and the average LS7 was 7.9 (2.3). Mortality from all-causes and CVD were 8.5% (4228/35,207) and 6.1% (2917/35,207), respectively. The median length of follow-up was 8.1 years. The combined burden of frailty and poor cardiovascular health on mortality risk varied according to age in males (FI*age interaction *p* = 0.01; LS7*age interaction *p* < 0.001) but not in females. In females, poor FI and LS7 combined to predict all-cause and CVD mortality in a dose-response manner. All-cause and CVD mortality risk was greater for older males (60 and 70 years old) who were at least mildly frail and had intermediate cardiovascular health or worse (hazard ratio [lower/higher confidence interval ranges] range: all-cause mortality = 2.02–5.30 [1.20–4.04, 3.15–6.94]; CVD-related mortality = 2.22–7.16 [1.03–4.46, 4.49–11.50]) but not for younger males (30, 40, and 50 years old).

**Conclusions:**

The combined burden of frailty and LS7 on mortality is similar across all ages in females. In males, this burden is greater among older people. Adding frailty to assessments of overall cardiovascular health may identify more individuals at risk for mortality and better inform decisions to implement preventative or treatment approaches.

**Supplementary Information:**

The online version contains supplementary material available at 10.1186/s12916-022-02593-w.

## Background

Poor cardiovascular health negatively impacts the quality of life and well-being of older adults [1] and independently increases the risk for incident cardiovascular disease (CVD) and CVD mortality [2–8]. In response, the American Heart Association (AHA) has suggested that risk reduction goals are needed to optimize cardiovascular health. The AHA defined cardiovascular health based on seven risk factors, including high cholesterol, blood pressure, glucose levels, smoking status, body mass index, low physical activity, and poor diet [9]. Huffman et al. introduced the Life’s Simple 7 (LS7) as a method to integrate these risk factors to define cardiovascular health into a single score to forecast cardiovascular outcomes [10, 11].

While the LS7 can inform disease prognosis, it was not designed to account for the burden of age-related health problems other than CVD. Given the rising global life expectancy [12, 13], understanding which individuals’ age in worse health is important when identifying those most at risk for adverse outcomes. Frailty as a measure of the accumulation of deficits can capture health problems at any age across the adult life course. It describes the variability in adverse health outcomes at a given age [14–16]. While there are several ways to measure and understand frailty, the two most common models are the frailty index (FI) [17] and the frailty phenotype [18]. The FI has been shown to increase with age [19]; it also predicts non-CVD mortality [20, 21], CVD mortality, and hospitalization [21–23]. FIs also perform similarly to the Framingham risk score (FRS) when discriminating CVD events [24] and has been used in adults over 20 years old [19, 25, 26]. In fact, high levels of frailty (as measured by FI or frailty phenotype) are associated with individual CVD risk factors [25, 27–31] and poor cardiovascular health [32]. Importantly, Farooqi et al. recently demonstrated that the combined burden of frailty and high CVD risk (measured using FRS) is associated with CVD events and CVD mortality [24]. However, the combined burden of cardiovascular risk factors and frailty on mortality is not well understood.

Sex-specific differences are also important in understanding the burden of poor cardiovascular health and frailty. For instance, females are two times more likely to be in ideal cardiovascular health than are males, and four more times more likely after adjusting for age, deprivation score, education, and depression [33]. Females also have higher frailty scores compared to males at all ages. However, males have higher mortality risk than exhibited by females with the same level of frailty [34]. Given the burden of poor cardiovascular health, more males are living with CVD and have a higher risk of dying from CVD compared to females [35]. This background motivates investigations into sex-specific differences in poor cardiovascular health and frailty. The objectives of this study were to examine for males and females separately, (1) the association between the LS7 and frailty, (2) if the LS7 and frailty predict all-cause and CVD-specific mortality independently, and (3) whether the combination of LS7 and frailty identifies more subgroups at risk for all-cause and CVD mortality than each on its own. This work quantifies mortality risk in relation to one’s overall health, cardiovascular health, and sex, and thus could better inform clinical decisions which manage the risk of mortality.

## Methods

### Study population

Data from nine cohorts of the National Health and Nutrition Examination Survey (1999-2015) were used. The NHANES database includes cross-sectional surveys of a nationally representative sample of non-institutionalized US residents [36, 37]. Data was downloaded from the website of the America Centers for Disease Control and Prevention (http://www.cdc.gov/nchs/nhanes.htm). The total sample of the NHANES 1999-2015 cohorts was 92,062. Our analysis sample included 35,207 participants after excluding people who were < 20 years of age (*n* = 42,550), had incomplete cardiovascular health information (*n* = 9,570), incomplete demographics information (*n* = 35), and insufficient data to create an FI (*n* = 5).

Each participant provided consent to participate in NHANES data collection. The NHANES protocol was approved by the institutional review board of the Centers for Disease Control and Prevention.

### Frailty index

Frailty was measured with a 33-item frailty index (33-FI) [17, 38] created using standard procedures [39]. The FI included deficits related to symptoms, signs, diseases, disabilities, and laboratory abnormalities. We used a modified version of a previously validated FI in NHANES (Additional file 1: Table S1) [19, 40, 41] by excluding items related to CVD (i.e. stroke, heart attack, high blood pressure, coronary heart disease), type-2 diabetes mellitus (i.e. glucose, hemoglobin A1C), and total cholesterol levels. Modification of FI to exclude items related to CVD has been previously validated [42]. The FI is the ratio of health deficits present, where scores theoretically range from 0 to 1. Participants were also divided into four frailty severity groups: non-frail (FI < 0.1), very mildly frail (0.1 ≤ FI < 0.2), mildly frail (0.2 ≤ FI < 0.3), and moderately/severely frail (0.3 ≤ FI) [40].

### Definition of Life’s Simple 7

The AHA developed a cardiovascular health metric using a combination of seven individual cardiovascular health metrics: smoking, BMI, physical activity, diet, total cholesterol, fasting glucose, and blood pressure [9]. Comprehensive details for the cardiovascular health behaviors and factors are included in Additional file 1. Based on previous literature, we combined these seven cardiovascular health metrics into a single Life’s Simple 7 Score (LS7) ranging from 0 to 14, with a higher score corresponding with better cardiovascular health [10]. These seven LS7 metrics were categorized as either poor (score = 0), intermediate (score = 1), or ideal (score = 2). The total LS7 was calculated per participant by summing their values. We also categorized participants into tertiles [43], with T1, T2, and T3 corresponding to 0-7, 8-9, 10-14 points on the LS7, respectively. T1 represent people with the worst cardiovascular health, T2 have intermediate cardiovascular health, and T3 represent those with the best cardiovascular health relative to the cohort. We included participants regardless of CVD history.

### Combined frailty and Life’s Simple 7 score groups

To evaluate the combined burden of frailty and LS7 on mortality, we joined the four frailty and LS7 groups. This resulted in 12 groups, with non-frail (FI < 0.1) and best cardiovascular health (T3 LS7) indicating the healthiest group. Additionally, to determine if combining the FI and the LS7 would result in greater mortality risk, we combined the seven individual LS7 health items (smoking, BMI, physical activity, diet, total cholesterol, fasting glucose, and blood pressure) [9] into the 33-item FI. This combined FI contains 40 items (33 original FI items + 7 LS7 items) and is referred to as the 40-FI. The 7 additional LS7 items are coded as 0 = ideal, 0.5 = intermediate, and 1 = poor according to their LS7 groupings.

### Mortality

Mortality status was examined with linked mortality certificate records from the National Death Index up until December 31, 2015. Survival time was counted from the date of the participants’ baseline examination center visit to the mortality event. All-cause and CVD-related mortality were analyzed. People who had an underlying leading cause of mortality as “disease of the heart,” “cerebrovascular disease,” or were flagged with hypertension as a cause of mortality were categorized as CVD-related mortality [44]. All other underlying causes of mortality were categorized as non-CVD related mortality and include mortality related to malignant neoplasms, chronic lower respiratory diseases, accidents, Alzheimer’s disease, diabetes, influenza and pneumonia, nephritis, nephrotic syndrome, and nephrosis.

### Statistical analysis

Demographic characteristics are presented as frequency (%) for categorical variables. Age was presented as mean ± standard deviation (SD). We compared inter-LS7 group differences with age and sex using chi-squared tests and analysis of variance. All regression models were stratified by sex. Multivariable linear regression was used to evaluate the association between individual cardiovascular health metrics and LS7 with FI scores (continuous); results were presented as *β*-coefficient with 95% confidence intervals (CI). We visualized the relationship between FI and LS7 by plotting the predicted values of FI from a linear regression model against LS7. We analyzed all-cause mortality risk across LS7 and FI (continuous and categorized scores) by using hazard ratios with 95% CI from Cox regression models; sub-distribution hazard ratios with the Fine-Gray model were used to evaluate CVD-related mortality risk (competing risk events). Non-CVD mortality analyses are reported in Additional file 1. Model 1 included the continuous versions of the 33-FI and the LS7, model 2 included the categorical versions of the 33-FI and the LS7, model 3 included the continuous version of the 40-FI, and model 4 included the categorical version of the 40-FI. We tested 2-way interactions between age and FI and age and LS7 for males and females. Simple slope analyses were performed when a significant interaction was present; age was centered at 30, 50, and 70 (ages 40 and 60 were also reported in Additional file 1). We accounted for the complex survey design and implemented survey weights provided by NHANES to all demographic statistics calculations and regression analyses apart from the Fine-Gray model. The healthiest FI level (FI < 0.1) and LS7 tertile (T3) were used as the reference group for all relevant regression models. All regression models were adjusted for age, education level, diagnosis of CVD, NHANES cycle number, and race. In addition, a sensitivity analysis to exclude all participants with a diagnosis of CVD (*n* = 3391; 7.57%) from the main regression models was performed and reported in Additional file 1. *p* values of < 0.05 were considered statistically significant. All data analyses were conducted using R version 4.0.5 and R Studio version 1.2.5 [45]. The “survey” package was used for all analyses on complex survey design data [46].

## Results

### Participant characteristics

The included participants (*n* = 35,207) had a mean age of 46.6 ± 16.7; 51.4% (*n* = 18,095) were female. People who were non-frail, very mildly frail, mildly frail, and moderately/severely frail had proportions of 70.4% (*n* = 22,538), 17.5% (*n* = 7084), 6.8% (*n* = 2983), and 5.3% (*n* = 2602), respectively. In both males and females, people with worse cardiovascular health (lower tertiles of LS7) were older (Table 1). Race other than White, Black, and Hispanic had the highest mean LS7 of all ethnicity categories (*p*-value < 0.01). The worst cardiovascular health group (T1 LS7) had the highest proportion of people that were smokers, had a poor diet, and higher biomarkers including BMI, total cholesterol, blood glucose, and blood pressure (Additional file 1: Table S2).

**Table 1:** Demographic statistics of all males and females by tertiles of Life’s Simple 7 score

		Males			Females		
LS7 tertile	***Total***	***3rd tertile***	***2nd tertile***	***1st tertile***	***3rd tertile***	***2nd tertile***	***1st tertile***
*(LS7 Score)*	*(0–14)*	*10–14*	*8–9*	*0–7*	*10–14*	*8–9*	*0–7*
**Sample size, *N***	35,207	3,827	5,549	7,736	4,860	5,577	7,658
**Age (mean ± SD)**	46.6 ± 16.7	38.2 ± 14.9	45.2 ± 15.9	51.5 ± 15.5	38.4 ± 14.0	46.8 ± 16.7	54.9 ± 15.9
**Female, *N*(%)**	18,095 (51.4%)	-	-	-	-	-	-
**CVD, *N*(%)**	3391 (7.57%)	156 (3.26%)	483 (6.54%)	1337 (13.88%)	90 (1.81%)	313 (4.88%)	1012 (11.73%)
**Race, *N*(%)**							
White	16,960 (70.8%)	1,853 (69.8%)	2,760 (71.7%)	3,756 (71.6%)	2,304 (69.9%)	2,620 (69.6%)	3,667 (71.8%)
Black	6,846 (10.3%)	696 (9.1%)	1,057 (9.4%)	1,555 (9.8%)	724 (8.2%)	1,067 (11.2%)	1,747 (13.2%)
Hispanic	9,101 (13.2%)	904 (14.0%)	1,377 (13.7%)	2,037 (13.7%)	1,329 (13.9%)	1,516 (13.5%)	1,938 (11.2%)
Other	2,300 (5.6%)	374 (7.1%)	355 (5.2%)	388 (4.8%)	503 (7.9%)	374 (5.7%)	306 (3.9%)
**Education, *N*(%)**							
< 9th grade	4,246 (5.9%)	275 (3.9%)	637 (5.7%)	1,268 (8.1%)	330 (3.5%)	615 (5.5%)	1,121 (7.5%)
9–11th grade	5,503 (12.1%)	466 (8.5%)	847 (11.7%)	1,392 (14.9%)	479 (6.9%)	815 (11.3%)	1,504 (16.7%)
High school	8,182 (24.0%)	803 (20.2%)	1,328 (24.8%)	1,937 (27.2%)	846 (16.3%)	1,329 (24.6%)	1,939 (28.2%)
Some college	9,829 (30.8%)	1,084 (29.2%)	1,450 (28.6%)	1,883 (29.1%)	1,538 (32.7%)	1,695 (33.5%)	2,179 (31.8%)
College graduate	7,447 (27.2%)	1,199 (38.2%)	1,287 (29.3%)	1,256 (20.8%)	1,667 (40.6%)	1,123 (25.0%)	915 (15.8%)
**Mortality rate, *N*(%)**							
All-cause	4,228 (8.5%)	259 (4.2%)	658 (7.2%)	1,491 (14.3%)	162 (2.2%)	475 (7.0%)	1,183 (12.7%)
CVD related	1,311 (2.4%)	57 (0.9%)	201 (2.0%)	499 (4.5%)	40 (0.5%)	118 (1.6%)	396 (3.8%)
Non-CVD related	2,917 (6.1%)	202 (3.3%)	457 (5.2%)	992 (9.8%)	122 (1.7%)	357 (5.4%)	787 (8.9%)
**Number of prescription medications, *N*(%)**							
8+	27,662 (80.82%)	3,605 (94.66%)	4,753 (88.28%)	5,369 (72.87%)	4,539 (92.66%)	4,630 (82.44%)	4,766 (62.69%)
4-7	5,711 (14.79%)	195 (4.57%)	663 (10.14%)	1714 (20.06%)	278 (6.57%)	786 (14.79%)	2,075 (26.66%)
0-3	1,813 (4.39%)	26 (0.77%)	129 (1.58%)	649 (7.06%)	40 (0.77%)	159 (2.77%)	810 (10.66%)
**33-Item Frailty Index, *N*(%)**							
< 0.1	22,538 (70.4%)	3,296 (89.2%)	4,089 (79.8%)	4,339 (64.3%)	3,978 (84.4%)	3,575 (67.3%)	3,261 (47.9%)
0.1–0.2	7,084 (17.5%)	387 (8.2%)	871 (13.2%)	1,711 (19.4%)	695 (12.1%)	1,316 (22.4%)	2,104 (25.7%)
0.2–0.3	2,983 (6.8%)	91 (1.7%)	359 (4.4%)	892 (8.8%)	128 (2.6%)	410 (6.4%)	1,103 (13.5%)
> 0.3	2,602 (5.3%)	53 (0.9%)	230 (2.5%)	794 (7.5%)	59 (0.8%)	276 (4.0%)	1,190 (12.9%)
Mean ± SD	0.09 ± 0.10	0.05 ± 0.06	0.07 ± 0.08	0.11 ± 0.11	0.06 ± 0.06	0.09 ± 0.09	0.15 ± 0.12

All percentages, means, and standard deviations are weighted. Higher LS7 tertiles indicate better cardiovascular health; lower Frailty Index indicate better overall health. *LS7* Life’s Simple 7 score, *CVD* cardiovascular disease, *SD* standard deviation

### Frailty is associated with cardiovascular health

The proportion of participants with better cardiovascular health (T3 LS7) was lower with higher frailty levels, from 30.3% and 40.1% in those who were non-frail to 5.5% and 4.1% in those who were moderately to severely frail for males and females, respectively (Additional file 1: Fig. S1). Multivariable linear regression revealed a significant age-by-33-FI interaction for cardiovascular health in both males and females (*p* < 0.01). Generally, a lower LS7 corresponded with higher 33-FI. In addition, a higher FI corresponded with a lower LS7 when age was centered at 70 compared to lower centered ages (30, 40, 50, and 60) (Additional file 1: Fig. S2). In addition, the 33-FI-LS7 slope was generally steeper (higher 33-FI per 1-point higher LS7) for females compared to males, especially at younger ages.

### Life’s Simple 7 score and frailty independently predict mortality

The total mortality rate was 8.5% (4223/30,930) (Table 1). The median length of follow-up was 97 months. Cox regression models predicting all-cause mortality showed a significant age-by-FI (*p* = 0.01) and age-by-LS7 (*p* < 0.001) interaction for males, but not for females (age-by-FI_female _*p* = 0.93; age-by-LS7_female _*p* = 0.54).

#### Females

A 0.01 greater 33-FI was associated with a 4% greater all-cause and 2% greater CVD-related mortality risk in females (Table 2). A 1-point higher LS7 (7% increase) was associated with a 5% lower all-cause and 9% lower CVD-related mortality risk. Using LS7 tertiles revealed that females with intermediate cardiovascular health (T1 LS7) did not have greater risk for CVD mortality when compared to females with the best cardiovascular health (T3 LS7) (Table 2).

**Table 2:** Association of frailty and cardiovascular health with mortality in females

Model	Term	Group	***N***	Mortality rate, ***N*** (%)		HR (95% CI)	SHR (95% CI)
				*All-cause*	*CVD-related*	*All-cause*	*CVD-related*
1	33-FI	Continuous	18,095	1,820 (7.7%)	554 (2.1%)	**1.04 (1.03,1.04)**	**1.02 (1.02,1.03)**
	LS7	Continuous	18,095	1,820 (7.7%)	554 (2.1%)	**0.95 (0.92,0.98)**	**0.91 (0.87,0.95)**
2	33-FI	0.0–0.1	10,814	421 (2.9%)	100 (0.6%)	Reference	
		0.1–0.2	4115	491 (10.3%)	153 (3.0%)	**1.49 (1.29,1.72)**	**1.47 (1.13,1.91)**
		0.2–0.3	1,641	392 (21.6%)	130 (6.3%)	**2.25 (1.87,2.70)**	**1.92 (1.45,2.56)**
		0.3<	1,525	516 (31.7%)	171 (9.6%)	**3.52 (2.94,4.21)**	**2.52 (1.91,3.32)**
	LS7	3rd tertile	4,860	162 (2.2%)	40 (0.5%)	Reference	
		2nd tertile	5,577	475 (7.0%)	118 (1.6%)	**1.40 (1.11,1.77)**	0.94 (0.66,1.35)
		1st tertile	7,658	1,183 (12.7%)	396 (3.8%)	**1.52 (1.21,1.89)**	**1.43 (1.02,2.01)**
3	40-FI	Continuous	18,095	1,820 (7.7%)	554 (2.1%)	**1.05 (1.04,1.05)**	**1.04 (1.03,1.04)**
4	40-FI	0.0–0.1	5,420	111 (1.4%)	21 (0.2%)	Reference	
		0.1–0.2	7,593	503 (5.4%)	138 (1.4%)	**1.54 (1.12,2.12)**	1.45 (0.90,2.31)
		0.2–0.3	2,921	513 (15.7%)	166 (4.7%)	**2.43 (1.76,3.35)**	**2.12 (1.31,3.45)**
		0.3<	2,161	693 (29.9%)	229 (8.9%)	**4.62 (3.24,6.59)**	**3.25 (2.00,5.27)**

Cox regression models were used for all-cause mortality; Fine-Gray models were used for CVD-related mortality. All models are adjusted for age, education level, diagnosis of CVD, NHANES cycle number, and race. Higher LS7 tertiles indicate better cardiovascular health, lower FI indicate better overall health. Hazard ratio for all-cause mortality is weighted; all mortality rate percentages are weighted. *HR* hazard ratio, *SHR* sub-distributional hazard ratio, *CI* confidence interval, *CVD* cardiovascular, *FI* frailty index, *LS7* Life’s Simple 7 score, *33-FI* FI with 33 items, *40-FI* 33-item FI combined with 7 items from the LS7. Bolded text indicates alpha < 0.05

#### Males

A 0.01 higher 33-FI was associated with a 2-4% greater risk of all-cause and CVD-related mortality, respectively, in males across all ages (Table 3). A 1-point higher LS7 was associated with a 6–20% lower risk of all-cause mortality and 10–25% lower risk of CVD-related mortality (Table 3). A 1-point higher LS7 in males at age 30 conferred greater associated reduction from all-cause and CVD-related mortality than a similar LS7 change in older males (Table 3). However, analyses using LS7 tertiles revealed that males with intermediate cardiovascular health (T2 LS7) did not have greater risk for all-cause and CVD mortality as compared to T3 LS7 across all ages.

**Table 3:** Association of frailty and cardiovascular health with mortality in males at ages 30, 50, and 70

Model	Term	Group	***N***	HR (95% CI) *All-cause mortality*			SHR (95% CI) *CVD-related mortality*		
				***Age 30***	***Age 50***	***Age 70***	***Age 30***	***Age 50***	***Age 70***
1	33-FI	Continuous	17,112	**1.02 (1.01,1.04)**	**1.03 (1.02,1.04)**	**1.04 (1.04,1.04)**	**1.04 (1.03,1.06)**	**1.03 (1.02,1.04)**	**1.02 (1.01,1.03)**
	LS7	Continuous		**0.80 (0.74,0.86)**	**0.87 (0.83,0.91)**	**0.94 (0.92,0.97)**	**0.75 (0.67,0.85)**	**0.83 (0.77,0.88)**	**0.90 (0.87,0.94)**
2	33-FI	0.0–0.1	11,724	Reference			Reference		
		0.1–0.2	2,969	1.21 (0.81,1.82)	**1.38 (1.11,1.71)**	**1.57 (1.38,1.78)**	**2.43 (1.29,4.58)**	**1.87 (1.32,2.65)**	**1.43 (1.18,1.75)**
		0.2–0.3	1,342	1.47 (0.79,2.75)	**1.76 (1.25,2.49)**	**2.11 (1.81,2.46)**	**3.62 (1.61,8.10)**	**2.33 (1.48,3.68)**	**1.50 (1.19,1.90)**
		0.3<	1,077	**2.72 (1.62,4.58)**	**3.25 (2.42,4.35)**	**3.87 (3.29,4.55)**	**5.93 (2.95,11.89)**	**3.66 (2.48,5.41)**	**2.26 (1.80,2.84)**
	LS7	3rd tertile	3,827	Reference			Reference		
		2nd tertile	5,549	0.99 (0.62,1.56)	0.96 (0.73,1.26)	0.94 (0.77,1.14)	1.06 (0.41,2.73)	1.20 (0.69,2.06)	**1.35 (1.01,1.82)**
		1st tertile	7,736	**2.39 (1.55,3.69)**	**1.69 (1.32,2.18)**	**1.20 (1.01,1.44)**	**2.91 (1.24,6.84)**	**2.26 (1.39,3.69)**	**1.76 (1.33,2.32)**
3	40-FI	Continuous	17,112	**1.03 (1.02,1.05)**	**1.04 (1.03,1.05)**	**1.05 (1.05,1.05)**	**1.05 (1.03,1.08)**	**1.04 (1.03,1.05)**	**1.02 (1.02,1.03)**
4	40-FI	0.0–0.1	5,901	Reference			Reference		
		0.1–0.2	7,275	1.23 (0.79,1.92)	**1.52 (1.19,1.94)**	**1.88 (1.45,2.45)**	1.35 (0.55,3.29)	1.56 (0.96,2.52)	**1.79 (1.15,2.80)**
		0.2–0.3	2,326	**1.84 (1.08,3.14)**	**2.36 (1.73,3.21)**	**3.01 (2.27,4.00)**	**3.09 (1.16,8.22)**	**2.74 (1.60,4.67)**	**2.42 (1.53,3.83)**
		0.3<	1,610	**2.83 (1.53,5.23)**	**4.02 (2.91,5.55)**	**5.69 (4.34,7.48)**	**7.58 (2.93,19.60)**	**5.19 (3.09,8.69)**	**3.55 (2.23,5.63)**

Cox regression models were used for all-cause mortality; Fine-Gray models were used for CVD-related mortality. All models are adjusted for age, education level, diagnosis of CVD, NHANES cycle number, and race. Higher LS7 tertiles indicate better cardiovascular health, lower FI indicate better overall health. Hazard ratios for all-cause mortality are weighted; all mortality rate percentages are weighted. *HR* hazard ratio, *SHR* sub-distributional hazard ratio, *CI* confidence interval, *CVD* cardiovascular, *FI* frailty index, *LS7* Life’s Simple 7 score, *33-FI* FI with 33 items, *40-FI* 33-item FI combined with 7 items from the LS7. Bolded text indicates alpha < 0.05. Mortality rates are available in Additional file 1: Table S9

### Combined burden of frailty and poor cardiovascular health on mortality

The combined burden of frailty and poor cardiovascular health on mortality risk varied with age in males (age-by-33-FI_male_ interaction *p* = 0.01; age-by-LS7_male_ interaction *p* < 0.001) but not in females (age-by-33-FI_female_ interaction *p* = 0.93; age-by-LS7_female_ interaction *p* = 0.54). These interactions were also observed when combining the 33-FI with LS7 metrics using the 40-FI (age-by-40-FI_male_ interaction *p* = 0.01, age-by-LS7_male_ interaction *p* < 0.001; age-by-40-FI_female_ interaction *p* = 0.91, age-by-LS7_female_ interaction *p* = 0.54).

#### Females

**Fig. 1:**
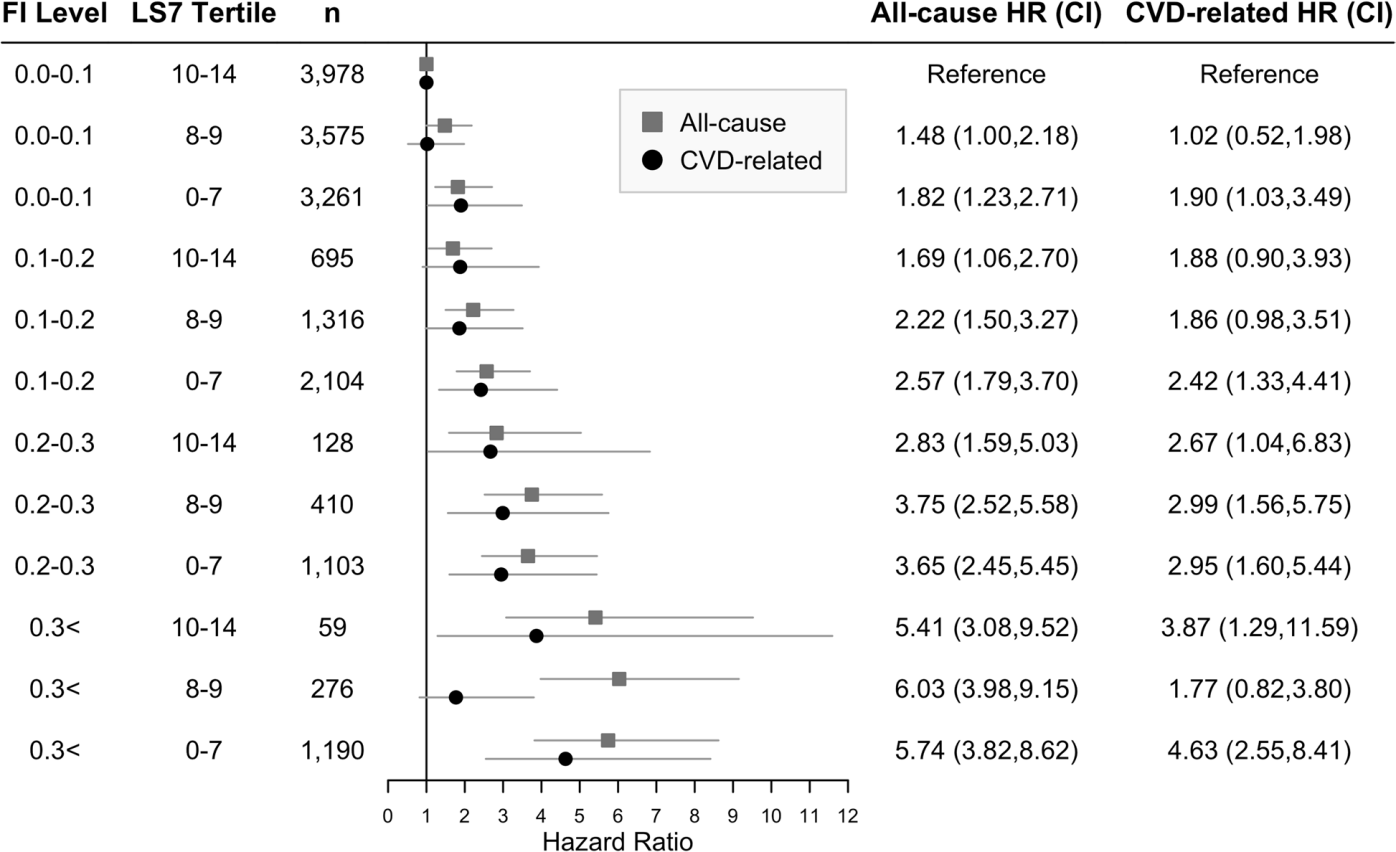
Cox regression and Fine-Gray models for combined effect of Life’s Simple 7 score and frailty on all-cause and CVD-related mortality in females. All models were adjusted for age, education level, diagnosis of CVD, NHANES cycle number, and race. The 95% confidence interval is indicated by gray lines. FI, frailty index; LS7, Life’s Simple 7 score; CVD, cardiovascular disease; CI, confidence interval; *n*, number of participants, *p > 0.05. LS7 for 3rd, 2nd, and 1st tertiles are 10–14, 8–9, and 0–7, respectively. The 0.0–0.1 FI and 3rd LS7 tertile represent the healthiest group, while the 0.3 < FI and 1st LS7 tertile represent the least healthy group; all other groups are intermediary between these two extremes. The 33-item FI was used in this forest plot

A 0.01 greater 40-FI was associated with a 5% greater all-cause and 4% greater CVD mortality risk in females (Table 2, model 3). The combined 33-FI and LS7 categories (12 categories) revealed a dose-response association with all-cause and CVD-related mortality risk across frailty and LS7 tertile groups (Fig. 1; Additional file 1: Table S3).

#### Males

**Fig. 2:**
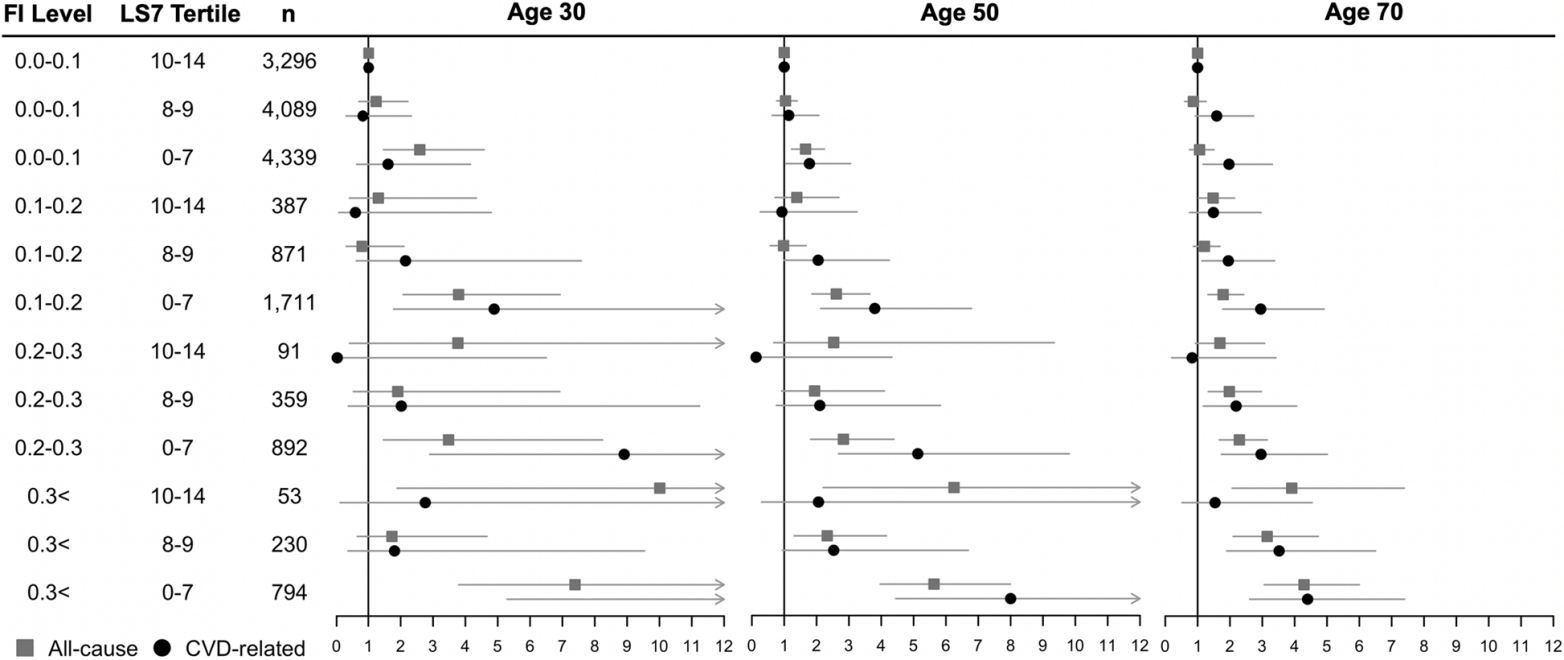
Cox regression and Fine-Gray models for combined effect of Life’s Simple 7 score and frailty on all-cause and CVD-related mortality in males, with age centered at 30, 50, and 70. All models were adjusted for age, education level, diagnosis of CVD, NHANES cycle number, and race. The 95% confidence interval is indicated by gray lines. FI, frailty index; LS7, Life’s Simple 7 score; CVD, cardiovascular disease; CI, confidence interval; *n*, number of participants, *p > 0.05. LS7 for 3rd, 2nd, and 1st tertiles are 10–14, 8–9, and 0–7, respectively. The 0.0–0.1 FI and 3rd LS7 tertile represent the healthiest group (reference group), while the 0.3 < FI and 1st LS7 tertile represent the least healthy group; all other groups are intermediary between these two extremes. The 33-item FI was used in this forest plot

A 0.01 higher 40-FI score was associated with a 3–5% greater all-cause and 2-5% greater CVD-related mortality in males across all ages (Table 3). However, using the combined 33-FI and LS7 categories identified additional subgroups at risk for mortality. For instance, males who had the worst frailty/cardiovascular health combination (0.3 < FI; T1 LS7) had higher risks for all-cause and CVD mortality when compared to the healthiest group (non-frail and T3 LS7) across all ages (Fig. 2; Additional file 1: Tables S4 and S5). All-cause and CVD mortality risk was greater for older males (60 and 70 years old) who were at least mildly frail (FI > 0.2) and had intermediate or worse cardiovascular health (T2/T3 LS7) (hazard ratio [confidence interval low, high]: all-cause mortality = 1.96 to 4.94 [1.17–3.71, 2.42–10.86]; CVD-related mortality = 2.14 to 5.93 [1.02–3.67, 4.06–9.59) but not for younger males (30, 40, and 50 years old) as compared to the healthiest group (non-frail and T3 LS7) (Fig. 2; Additional file 1: Tables S4 and S5). Overall, the combined effect of poor cardiovascular health and high frailty levels on mortality risk was mitigated at a younger age.

### Sensitivity analysis

Exclusion of participants with a diagnosis of CVD (*n* = 3,391) did not significantly change results (Additional file 1: Tables S6 and S7, Figs. S3 and S4). There was still a dose-response association between 33-FI and LS7 categories with all-cause and CVD-related mortality risk in females. In addition, the combined effect of poor cardiovascular health and high frailty levels on mortality risk was similarly mitigated for younger aged males.

## Discussion

### Summary of results

This study investigated the relationship between cardiovascular health and frailty as an accumulation of non-cardiovascular deficits on mortality risk in females and males across age. We found that poor cardiovascular health was associated with higher frailty in both females and males; this effect was more pronounced in older people (objective 1). Generally, people with higher frailty or worse cardiovascular health had a higher risk of all-cause and CVD-related mortality (objective 2). Lastly, the combination of poor cardiovascular health and a higher frailty burden predicts greater all-cause and CVD-related mortality risk. Females with greater degrees of frailty and poor cardiovascular health had at higher mortality risk relative to their healthy peers at all ages. In males, this burden was worse at older ages (objective 3). Here, we elucidated the implications of non-cardiovascular deficits accumulation and cardiovascular health on mortality; these findings provide new information on a patient’s mortality risk by sex and age.

### Frailty and cardiovascular health

Here, we demonstrate that the relationship between frailty and cardiovascular health differs by age in both males and females. At similar frailty levels, older males and females had worse cardiovascular health compared to their younger peers. We also demonstrated that individual cardiovascular risk factors including poor smoking status, BMI, physical activity level, fasting blood glucose, and blood pressure were related to higher frailty levels (Additional file 1: Fig. S5), which is in agreement with previous reports [25, 29, 31, 47, 48]. These findings in combination with previous work linking high CVD risk with the onset of frailty (phenotype [49] and FI [50]) and subclinical CVD markers [51, 52] with frailty further corroborate the intertwined nature of CVD and frailty. Indeed, the relationship between frailty and cardiovascular health may have important clinical implications before a CVD diagnosis.

In addition, our study makes a novel contribution by demonstrating that frailty as an accumulation of non-cardiovascular deficits is related to cardiovascular health and that this relationship differs with age in males and females, aligning with a body of work suggesting that the problems of old age come as a package [53]. This result highlights the role of age-related physiologic systems not directly related to cardiovascular problems. In consequence, not only is age important in describing this relationship, but as others have observed, so is understanding the degree of frailty in relation to how cardiovascular health and its associated adverse outcomes change with age [21, 24, 53–55].

### Frailty and cardiovascular health independently predicts mortality

Here, we add to the existing literature that frailty and cardiovascular health are independently related to all-cause and CVD-related mortality risk (Tables 2 and 3) [19, 21, 24, 43, 56–60]. Our FI, which did not include items related cardiovascular health, was associated CVD-related mortality for both females and males across all ages; this aligns with previous studies [22, 24, 61, 62]. Together, these results show that non-cardiovascular items or risk factors predict CVD-related mortality when indexed in the context of deficit accumulation.

### Sex and the combined burden of frailty and cardiovascular health

We showed that for females, the combined burden of frailty and poor cardiovascular health resulted in greater mortality risk uniformly with higher frailty levels and worse cardiovascular health irrespective of age (Fig. 1; Table 2). In males, the effect of frailty and poor cardiovascular health on all-cause mortality risk was greater at older versus younger age. Specifically, the association with all-cause mortality of the 40-FI appear greater than the 33-FI especially at older ages (Fig. 2; Table 3). The 40-FI demonstrates that adding the LS7 to an FI can incrementally increase the magnitude of the FI’s association with mortality; adding other health items to an FI may not yield a similar effect as that of the LS7.

These results are relevant when considering how interventions may affect different populations, particularly males of different ages. For example, our data motivates further study to determine if younger males who live with mild frailty and poor cardiovascular health could have greater mortality risk reductions if they improve their cardiovascular health before older age. In females, improving cardiovascular health or frailty regardless of age could reduce their mortality risk. Nevertheless, whether females or males will derive a greater benefit from managing frailty or improving cardiovascular health require further investigation.

### Combination of frailty and cardiovascular health in relation to mortality

We showed that greater degrees of frailty in combination with worse cardiovascular health exacerbates mortality risk (Figs. 1 and 2; Tables 2 and 3), thus demonstrating frailty’s added prognostic value to overall cardiovascular health when examining mortality risk (irrespective of causes). This finding is similar to a previous study [22], which highlighted that deficits not related to the AHA’s definition of cardiovascular health is also important for evaluating the risk of adverse events associated with cardiovascular health. Furthermore, the result that frailty status helps to identify new subgroups at risk among people with similar cardiovascular health is concordant with a previous study showing that an FI and the Framingham risk score have additive information for discriminating CVD events (C-statistic of FI, Framingham risk score, and both together are 0.60, 0.58, and 0.66, respectively) in an older population (mean age, 70.8 years) [24]. Together, these data are relevant as we consider the AHA’s goal to reduce mortality from CVD [9]—accurate identification of individuals at risk is crucial for appropriate and efficient delivery of any interventions. This knowledge motivates further inquiry as to whether the consideration of frailty alongside overall cardiovascular health in clinical settings will enable better management of patient cardiovascular health. Specifically, future work should investigate if the addition of frailty tools (Clinical Frailty Scale, frailty phenotype, or FI) will improve mortality predictions by cardiovascular health scores. This idea could be realized by harnessing electronic medical record data routinely collected as part of standard care for patients to develop a FI [63, 64].

In this context, we hypothesize that treatments to either improve cardiovascular health or manage frailty may also incrementally improve patient health outcomes and lower cardiovascular risk. A recent study from our research group showed that cardiac rehabilitation completion was associated with lower frailty levels in two thirds of patients [65]. This frailty reduction effect, alongside improvements in CVD risk factors, suggest that interventions which model multidisciplinary exercise and education-based cardiac rehabilitation programs could be an effective treatment strategy for frail patients with poor cardiovascular health. Indeed, this invites further inquiry to study cardiac rehabilitation and its effect on long-term health as both a primary and secondary prevention measure of CVD and subsequent management of frailty. In addition, the AHA has recently updated the LS7 to the Life’s Essential 8, adding a new “sleep health” component to the construct of cardiovascular health [66]. Future research should also evaluate the role of sleep with previous cardiovascular health metrics in relation to frailty and mortality.

### Strengths and limitations

A strength of our study was that we used a large and robust study cohort of 35,207 individuals, of which are nationally representative of community-dwelling US adults, with long-term follow-up. However, our data have limitations. First, despite the NHANES being a complex, multistage, and rigorous survey, the baseline data of non-institutionalized United States of America population are cross-sectional and thus we cannot examine the causal nature of relationship between frailty and cardiovascular health. Furthermore, since the NHANES used self-reported measures of physical activity, smoking, and various other items used to create the FI and LS7, classification errors or recall bias can operate when responding to surveys; however, self-report survey use in frailty indices has been validated [67]. It is also important to note that we only used complete cases of data for the creation of the LS7. Any participant missing 1 or more of the 7 cardiovascular health metrics were excluded (*n* = 9570) as creation of the LS7 requires availability of all seven cardiovascular health metrics. Participants with incomplete data were often older and frailer (Additional file 1: Table S8). As such, these data may have biased prevalence estimates of demographic and mortality data. Additionally, mortality may not be the most robust outcome for younger adults. The wide confidence intervals and large hazard ratios (Fig. 2; Additional file 1: Tables S4 to S5) for some groups of young males may be attributable to low sample sizes and mortality events; the paradoxical nature of being severely frail but concurrently having good cardiovascular health seems to have resulted in a scarcity of data in this young population.

## Conclusions

Our study revealed that frailty as an accumulation of non-cardiovascular deficits is related to overall cardiovascular health in both females and males. Generally, females and males with higher frailty or worse cardiovascular health are more likely to die. The combined burden of frailty and poor cardiovascular health on mortality is higher in a dose-response trend for females. For males, the lethality of this combined burden is greater in older males than in younger males. Adding frailty to assessments of overall cardiovascular health may identify more individuals at risk for mortality and thus has the potential to improve decisions to implement preventative or treatment approaches.

## Supplementary Information

**Additional file 1. **Expanded Methods. Additional details for assessments of cardiovascular health behaviours and factors. Expanded Results. Additional results for individual LS7 metrics and non-CVD mortality. Expanded Discussion. Additional discussion for non-CVD mortality. **Table S1.** 33-item frailty index. **Table S2.** Cardiovascular health behaviors and factors by tertiles of Life’s Simple 7 score and sex. **Table S3.** Combined effect of frailty and cardiovascular health on mortality in females. **Table S4.** Combined effect of FI and LS7 on all-cause mortality in males across ages. **Table S5.** Combined effect of FI and LS7 on CVD mortality in males across ages. **Table S6.** Association of frailty and cardiovascular health with mortality in females without a CVD diagnosis. **Table S7.** Association of frailty and cardiovascular health with mortality in males without a CVD diagnosis at ages 30, 50, and 70. **Table S8.** Characteristics of participants excluded due to incomplete cardiovascular information. **Table S9.** Mortality rates by frailty and Life’s Simple 7 score groups in males. **Table S10.** Association of frailty and cardiovascular health with non-CVD mortality in females. **Table S11.** Associations of frailty and cardiovascular health with non-CVD mortality in males at ages 30, 50, and 70. **Table S12.** Combined effect of FI and LS7 on non-CVD mortality in males across ages. **Table S13.** Demographic statistics of all males and females by age groups. **Table S14.** Cardiovascular health behaviors and factors by age groups. **Figure S1.** Proportion of participants in each Life’s Simple 7 score tertile by frailty index level (33-item version) for males and females. **Figure S2.** Simple slopes of the association between Life’s Simple 7 score and the 33-item frailty index from a linear regression model for males and females. **Figure S3.** Cox regression and Fine-Gray models for combined effect of Life’s Simple 7 score and frailty on all-cause and CVD-related mortality in females without a CVD diagnosis. **Figure S4.** Cox regression and Fine-Gray models for combined effect of Life’s Simple 7 score and frailty on all-cause and CVD-related mortality in males without a CVD diagnosis, with age centered at 30, 50, and 70. **Figure S5.** Multiple linear regression model for the association between individual cardiovascular health metrics and frailty.

## Data Availability

This dataset used is publicly available from https://www.cdc.gov/nchs/nhanes/index.htm.

## Abbreviations

AHA: American Heart Association
BMI: Body mass index
LS7: Cardiovascular health score
CVD: Cardiovascular disease
FI: Frailty index
NHANES: National Health and Nutrition Examination Survey
SD: Standard deviation
T1 LS7: First tertile of the cardiovascular health score
T2 LS7: Second tertile of the cardiovascular health score
T3 LS7: Third tertile of the cardiovascular health score
