# Supplementary material for: The impact of cardiovascular health and frailty on mortality for males and females across the life course
Source: BMC Med. 2022 Nov 11;20:394. doi: 10.1186/s12916-022-02593-w (PMC9650802; doi:10.1186/s12916-022-02593-w)
Supplement: Supplementary file 1 — Additional file 1. Expanded Methods. Additional details for assessments of cardiovascular health behaviours and factors. Expanded Results. Additional results for individual LS7 metrics and non-CVD mortality. Expanded Discussion. Additional discussion for non-CVD mortality. Table S1. 33-item frailty index. Table S2. Cardiovascular health behaviors and factors by tertiles of Life’s Simple 7 score and sex. Table S3. Combined effect of frailty and cardiovascular health on mortality in females. Table S4. Combined effect of FI and LS7 on all-cause mortality in males across ages. Table S5. Combined effect of FI and LS7 on CVD mortality in males across ages. Table S6. Association of frailty and cardiovascular health with mortality in females without a CVD diagnosis. Table S7. Association of frailty and cardiovascular health with mortality in males without a CVD diagnosis at ages 30, 50, and 70. Table S8. Characteristics of participants excluded due to incomplete cardiovascular information. Table S9. Mortality rates by frailty and Life’s Simple 7 score groups in males. Table S10. Association of frailty and cardiovascular health with non-CVD mortality in females. Table S11. Associations of frailty and cardiovascular health with non-CVD mortality in males at ages 30, 50, and 70. Table S12. Combined effect of FI and LS7 on non-CVD mortality in males across ages. Table S13. Demographic statistics of all males and females by age groups. Table S14. Cardiovascular health behaviors and factors by age groups. Figure S1. Proportion of participants in each Life’s Simple 7 score tertile by frailty index level (33-item version) for males and females. Figure S2. Simple slopes of the association between Life’s Simple 7 score and the 33-item frailty index from a linear regression model for males and females. Figure S3. Cox regression and Fine-Gray models for combined effect of Life’s Simple 7 score and frailty on all-cause and CVD-related mortality in females without a CVD diagnosis. Figure [file 12916_2022_2593_MOESM1_ESM.docx]

# Additional File 1

## Expanded Methods

### Assessment of Cardiovascular Health Behaviors

We determined smoking status based on responses to the following questions, “Do you now smoke cigarettes?” and “How long has it been since you quit smoking cigarettes?” Current smokers are categorized to as ‘poor’ cardiovascular health. Former smokers who quit less than or 12 months ago were categorized as ‘intermediate’ cardiovascular health and those quit more than 12 months ago or have never smoked before were categorized as ‘ideal’ cardiovascular health.

Height and weight were measured in with standardized equipment and techniques. We calculated body mass index by dividing weight in kilograms by height in meters squared. People with BMIs that were ≥30.0 kg/m^2^, 25.0-29.9kg/m^2^, or <25.0 kg/m^2^ were categorized as ‘poor’, ‘intermediate’, and ‘ideal’, respectively.

We determined physical activity level based on responses the following questions about the frequency and duration of physical activity, “Over the past 30 days, what moderate activities did you do?” and “Over the past 30 days, how often did you perform those activities?” Similar questions were asked for vigorous activities. People who reported zero minutes/week of moderate and vigorous physical activity were categorized as ‘poor’, people with 1-149 minutes/week were categorized as ‘intermediate’, and those with more than 150 minutes/week were categorized as ‘ideal’.

We determined the diet of participants through a combination of food frequency questionnaire and 24-hour dietary recall interviews. From these interviews, the proportion of U.S. adults who meet the primary five goals of the Healthy Diet Score (HDS) used to define cardiovascular health were assessed. The five goals of the HDS are: (1) fruits and vegetables ≥ 4.5 cups/day, (2) fish ≥ two 3.5-ounce servings/week, (3) whole grains ≥ three 1 oz. servings/day, (4) sodium < 1500 mg/day, and (5) added sugar in sugar-sweetened beverages < 450 kcal/week. A 2,000 kcal/day diet was used to scale intakes of fruits, vegetables, fish, and whole grains. We followed the methodology established by the USDA Center for Nutrition Policy and Promotion to calculate all dietary intake factors.^58^ People who met either 0-1, 2-3, or 4-5 goals of the HDS were categorized as ‘poor’, ‘intermediate’, and ‘ideal’, respectively.

### Assessment of Cardiovascular Health Factors

Total cholesterol, and fasting blood glucose were determined by analysis of blood samples at centralized laboratories. Participated were requested to fast for more than 12 hours before blood sample collection. The Mobile Examination Clinic Laboratory Procedures Manual^59^ provides in-depth detail on the blood collection and processing procedures.

In addition to total cholesterol measurements, we determined whether participants received treatments for high cholesterol from the question, “To lower your blood cholesterol, have you ever been told by a doctor or other health professional to take prescribed medicine?” followed by “Are you now following this advice to take prescribed medicine?”. People with total cholesterol level of either <200 mg/dL untreated, 200-239 mg/dL or treated to goal, or ≥ 240 ml/dL were categorized as ‘poor’, ‘intermediate’, and ‘ideal’, respectively.

Similarly, we determined treatment status for high fasting blood glucose by responses to the questions: “Are you now taking insulin?” and “Are you now taking diabetes pills to lower your blood sugar?” People with fasting blood glucose level of either <100 mg/dL untreated, 100-125 mg/dL or treated to goal, or ≥ 126 ml/dL were categorized as ‘poor’, ‘intermediate’, and ‘ideal’, respectively.

Systolic (SBP) and diastolic (DBP) blood pressure were measured after the participant had rested for 5 minutes in a sitting position with both feet on the floor. Blood pressure was measured at most three times. We used the mean of the last two measurements if there were three measurements, and the last measurement if there were two or one. We determined the status of blood pressure treatment from the question, “Because of your hypertension/high blood pressure, have you ever been told to take a prescribed medicine?” followed by, “Are you now taking a prescribed medicine?” People with blood pressures of SBP <120/DBP<80 untreated, SBP 120-139 or DBP 80-89 or treated to goal, or SBP ≥ 140 or DBP ≥ 90 or treated to goal were categorized as ‘poor’, ‘intermediate’, and ‘ideal’, respectively.

## Expanded Results

### Individual Life’s Simple 7 Score Metrics and Frailty

We also examined the association of the seven individual LS7 metrics with frailty (Figure S5). Linear regression models adjusted for covariates revealed that compared with people in each respective ‘ideal’ category, those with ‘poor’ smoking status, BMI, physical activity level, cholesterol level, fasting blood glucose, and blood pressure had significantly higher FI scores. Healthy Diet Score was not associated with frailty in any category. By contrast, ‘poor’ cholesterol and ‘intermediate’ BMI each were significantly associated with lower FI scores when compared to their respective ideal category.

### Frailty, Cardiovascular Health, and Non-Cardiovascular Disease Mortality

Cardiovascular health was associated with non-CVD mortality in all females (Table S10), and males at 30 and 50 years old (Table S11). When using LS7 tertiles, there were no tertiles that showed greater non-CVD mortality risk in both females and males. The 33-FI was associated with non-CVD mortality risk in females and males of all ages (Tables S8 and S9). However, males at ages 30 and 50 did not have greater non-CVD mortality if they were very mildly frail when compared to non-frail peers (Table S11). Using the 40-FI, females and males had greater non-CVD mortality risk for every 0.01 increase in the FI score compared to the 33-FI. For females, the combination of FI and LS7 categories also had a dose-response effect on non-CVD related mortality, but to a lesser extent than all-cause and CVD mortality (Table S3). When compared to the healthiest group (non-frail and T3 LS7), males who were moderately/severely frail and had intermediate cardiovascular health or worse had greater risk for non-CVD mortality across all age groups (Table S12). Like all-cause and CVD mortality, the combined burden of frailty and poor cardiovascular health was lower for younger males and affects females of all ages similarly.

## Expanded Discussion

### Non-CVD related mortality risk

Our 33-item FI aligns with previous work^20^ as it is associated with non-CVD related mortality risk in both females (Table S3) and males (Tables S4). The 40-item Combined-FI and the combination of frailty and cardiovascular health categories showed that using the FI can help the LS7 identify more subgroups at risk for mortality not related to CVD, and vice versa.

##

## Supplementary Tables

##### **Table S1.** 33-item frailty index.

| **Self-reported items** | **Lab items (normal range)** |
| --- | --- |
| Thyroid condition | Mean cell volume (80-96 fL) |
| Cancer | Platelet count SI (150,000-450,000 cells/µL) |
| Arthritis | Sodium (136-142 mmol/L) |
| Weak/failing kidneys | Blood urea nitrogen (3-20 mg/dL) |
| Confusion/inability to remember | Bicarbonate (≤28 mmol/L) |
| Difficulty managing money | Red cell distribution width (≤14.6%) |
| Difficulty stooping, crouching, kneeling | Lactate dehydrogenase (≤190 U/L) |
| Difficulty lifting or carrying | Alkaline phosphatase (≤115 U/L) |
| Difficulty preparing meals | Creatinine (M: 60-110, F: 45-90 µmol/L) |
| Difficulty walking between rooms on same floor | Hemoglobin (M: 13.5-18, F: 12-16 g/dL) |
| Difficulty standing up from armless chair | Uric acid (M: 240-510, F: 160-430 µmol/L) |
| Difficulty getting in and out of bed | Total calcium (2-2.5 mmol/L) |
| Difficulty using fork and knife |  |
| Difficulty dressing yourself difficulty |  |
| Difficulty grasping/holding small objects |  |
| Difficulty attending social event |  |
| Self-reported health |  |
| Frequency of healthcare use |  |
| Health compared to 1 year ago |  |
| Overnight hospital stays |  |
| Medications |  |

*M* male*, F* female

##### **Table S2.** Cardiovascular health behaviors and factors by tertiles of Life’s Simple 7 score and sex.

|  |  | **Males** | | |  | **Females** | | |
| --- | --- | --- | --- | --- | --- | --- | --- | --- |
| **LS7 Tertile** | ***Total*** | ***3^rd^ tertile*** | ***2^nd^ tertile*** | ***1^st^ tertile*** |  | ***3^rd^ tertile*** | ***2^nd^ tertile*** | ***1^st^ tertile*** |
| *(LS7 Score)* | *0-14* | *10-14* | *8-9* | *0-7* |  | *10-14* | *8-9* | *0-7* |
| **Sample Size**, *N* | 35,207 | 3,827 | 5,549 | 7,736 |  | 4,860 | 5,577 | 7,658 |
| **Smoking Status**, *N (%)* | | | | | | | | |
| Never or quit > 12 months prior | 18,761 (52.8%) | 2,817 (73.4%) | 2,626 (48.0%) | 2,040 (26.7%) |  | 4,024 (79.1%) | 3,633 (59.2%) | 3,621 (43.0%) |
| Former, quit ≤ 12 months prior | 8,920 (25.0%) | 613 (16.9%) | 1,618 (27.3%) | 3,095 (37.9%) |  | 551 (13.9%) | 1,027 (20.7%) | 2,016 (27.8%) |
| Current Smoker | 7,526 (22.2%) | 397 (9.7%) | 1,305 (24.7%) | 2,601 (35.4%) |  | 285 (7.0%) | 917 (20.0%) | 2,021 (29.2%) |
| **Body Mass Index** (kg/m^2^), *N (%)* | | | | | | | | |
| <25.0 | 10,535 (31.9%) | 2,185 (55.3%) | 1,689 (27.2%) | 978 (10.6%) |  | 3,115 (68.4%) | 1,658 (31.9%) | 910 (12.6%) |
| 25.0-29.9 | 12,165 (34.1%) | 1,384 (37.9%) | 2,478 (45.7%) | 2,923 (36.2%) |  | 1,280 (23.8%) | 1,989 (35.0%) | 2,111 (27.6%) |
| ≥30.0 | 12,507 (34.0%) | 258 (6.8%) | 1,382 (27.1%) | 3,835 (53.3%) |  | 465 (7.8%) | 1,930 (33.1%) | 4,637 (59.8%) |
| Mean ± SD | 28.5 ± 6.5 | 25.1 ± 3.9 | 28.0 ± 5.2 | 31.0 ± 6.1 |  | 4.2 ± 4.5 | 28.5 ± 6.6 | 32.2 ± 7.4 |
| **Physical Activity** (minutes/week), *N (%)* | | | | | | | | |
| 150 moderate or vigorous activity | 15,634 (49.5%) | 3,062 (81.7%) | 3,372 (64.8%) | 2,346 (34.%) |  | 3,195 (69.2%) | 2,303 (44.6%) | 1,356 (19.2%) |
| 1-149 moderate or vigorous activity | 8,804 (25.0%) | 578 (14.6%) | 1,306 (23.1%) | 2,082 (27.8%) |  | 1,140 (22.3%) | 1,726 (31.1%) | 1,972 (27.4%) |
| No activity | 10,769 (25.5%) | 187 (3.7%) | 871 (12.1%) | 3,308 (37.8%) |  | 525 (8.5%) | 1,548 (24.3%) | 4,330 (53.3%) |
| Mean ± SD | 332.4 ± 513.4 | 578.5 ± 632.1 | 481.0 ± 594.4 | 265.2 ± 521.8 |  | 386.0 ± 454.0 | 271.2 ± 432.1 | 123.7 ± 301.4 |
| **Healthy Diet Score†**, *N (%)* | | | | | | | | |
| 4-5 goals met | 520 (1.2%) | 95 (1.7%) | 63 (0.7%) | 42 (0.4%) |  | 160 (2.9%) | 100 (1.3%) | 60 (0.6%) |
| 2-3 goals met | 12,696 (34.7%) | 1,783 (45.1%) | 1,983 (32.4%) | 2,229 (26.1%) |  | 2,373 (47.9%) | 2,108 (36.0%) | 2,220 (26.8%) |
| 0-1 goals met | 21,991 (64.1%) | 1,949 (53.2%) | 3,503 (66.9%) | 5,465 (73.5%) |  | 2,327 (49.2%) | 3,369 (62.7%) | 5,378 (72.6%) |
| **Total Cholesterol** (mg/dL), *N (%)* | | | | | | | | |
| <200 untreated | 15,965 (46.1%) | 2,969 (76.7%) | 2,863 (49.4%) | 2,152 (25.4%) |  | 3,579 (73.3%) | 2,566 (45.4%) | 1,836 (23.0%) |
| 200-239/treated to goal | 13,946 (39.1%) | 796 (21.4%) | 2,240 (41.7%) | 3,847 (49.7%) |  | 1,107 (23.4%) | 2,228 (41.0%) | 3,728 (48.7%) |
| ≥240 | 5,296 (14.8%) | 62 (1.8%) | 446 (8.9%) | 1,737 (24.9%) |  | 174 (3.3%) | 783 (13.6%) | 2,094 (28.2%) |
| Mean ± SD | 197.7 ± 41.7 | 178.3 ± 31.5 | 192.9 ± 36.8 | 207.5 ± 47.3 |  | 182.6 ± 30.6 | 199.9 ± 39.2 | 214.8 ± 45.0 |
| **Fasting Blood Glucose** (mg/dL), *N (%)* | | | | | | | | |
| <100 untreated | 24,118 (73.0%) | 3,506 (92.6%) | 4,181 (78.2%) | 3,379 (48.3%) |  | 4,628 (95.5%) | 4,598 (83.8%) | 3,826 (53.6%) |
| 100-125 or treated to goal | 7,639 (19.6%) | 300 (7.0%) | 1,166 (19.1%) | 2,734 (34.2%) |  | 219 (4.2%) | 848 (14.0%) | 2,372 (30.4%) |
| ≥126 | 3,450 (7.4%) | 21 (0.4%) | 202 (2.7%) | 1,623 (17.4%) |  | 13 (0.2%) | 131 (2.1%) | 1,460 (15.9%) |
| Mean ± SD | 97.5 ± 31.9 | 87.9 ± 11.5 | 93.6 ± 20.0 | 111.2 ± 44.5 |  | 85.5 ± 10.7 | 90.7 ± 17.6 | 108.2 ± 43.7 |
| **Blood Pressure** (mmHg), *N (%)* | | | | | | | | |
| SBP<120 or DBP<80 untreated. | 13,417 (41.4%) | 2,486 (66.9%) | 1,883 (35.1%) | 969 (14.2%) |  | 3,968 (81.0%) | 2,750 (48.9%) | 1,361 (19.1%) |
| SBP 120-139 \| DBP 80-89 \| treated to goal. | 14,738 (42.2%) | 1,232 (30.9%) | 2,928 (54.0%) | 4,075 (55.1%) |  | 774 (16.9%) | 2,110 (39.5%) | 3,619 (49.6%) |
| SBP ≥140 or DBP ≥90 | 7,052 (16.4%) | 109 (2.1%) | 738 (10.9%) | 2,692 (30.7%) |  | 118 (2.1%) | 717 (11.6%) | 2,678 (31.3%) |
| Mean SBP ± SD | 122.2 ± 17.7 | 115.5 ± 10.4 | 122.1 ± 13.5 | 130.0 ± 17.0 |  | 110.0 ± 12.1 | 119.6 ± 17.7 | 130.5 ± 20.7 |
| Mean DBP ± SD | 70.8 ± 12.4 | 68.6 ± 10.3 | 72.4 ± 11.5 | 74.7 ± 13.8 |  | 67.2 ± 9.3 | 69.6 ± 11.8 | 71.0 ± 14.2 |

All percentages, means, standard deviations are weighted.

Higher LS7 tertiles indicate better cardiovascular health.

†The five Healthy Diet Score goals are: (1) fruits and vegetables ≥ 4.5 cups/day, (2) fish ≥ two 3.5-ounce servings/week, (3) whole grains ≥ three 1 oz. servings/day, (4) sodium < 1500 mg/day, and (5) added sugar in sugar-sweetened beverages < 450 kcal/week.

LS7 = Life’s Simple 7 score, DBP = diastolic blood pressure, SBP = systolic blood pressure, SD = standard deviation.

##### **Table S3.** Combined effect of frailty and cardiovascular health on mortality in females.

| **FI** | **LS7** | **Number of Deaths, N (%)** | | |  | **HR (95% CI)** | **SHR (95% CI)** | | |
| --- | --- | --- | --- | --- | --- | --- | --- | --- | --- |
|  |  | *All-cause* | *CV related* | *Non-CV related* |  | *All-cause* | *CV related* | *Non-CV related* |  |
| 0.0-0.1 | 10-14 | 70 (1.20%) | 13 (0.15%) | 57 (1.05%) |  | Reference | | |  |
| 0.0-0.1 | 8-9 | 138 (3.11%) | 25 (0.44%) | 113 (2.66%) |  | 1.48 (1.00,2.18) | 1.02 (0.52,1.98) | 1.34 (0.97,1.84) |  |
| 0.0-0.1 | 0-7 | 213 (4.99%) | 62 (1.35%) | 151 (3.64%) |  | **1.82 (1.23,2.71)** | **1.90 (1.03,3.49)** | **1.48 (1.09,2.02)** |  |
| 0.1-0.2 | 10-14 | 53 (5.40%) | 15 (1.66%) | 38 (3.74%) |  | **1.69 (1.06,2.70)** | **1.88 (0.90,3.93)** | **1.69 (1.11,2.56)** |  |
| 0.1-0.2 | 8-9 | 138 (9.37%) | 40 (2.25%) | 98 (7.12%) |  | **2.22 (1.50,3.27)** | **1.86 (0.98,3.51)** | **1.74 (1.24,2.43)** |  |
| 0.1-0.2 | 0-7 | 300 (12.79%) | 98 (4.14%) | 202 (8.65%) |  | **2.57 (1.79,3.70)** | **2.42 (1.33,4.41)** | **1.98 (1.46,2.70)** |  |
| 0.2-0.3 | 10-14 | 23 (13.76%) | 7 (3.50%) | 16 (10.27%) |  | **2.83 (1.59,5.03)** | **2.67 (1.04,6.83)** | **2.54 (1.47,4.40)** |  |
| 0.2-0.3 | 8-9 | 106 (24.88%) | 36 (8.52%) | 70 (16.36%) |  | **3.75 (2.52,5.58)** | **2.99 (1.56,5.75)** | **2.63 (1.83,3.79)** |  |
| 0.2-0.3 | 0-7 | 263 (21.63%) | 87 (5.96%) | 176 (15.67%) |  | **3.65 (2.45,5.45)** | **2.95 (1.60,5.44)** | **2.78 (2.02,3.83)** |  |
| 0.3< | 10-14 | 16 (23.11%) | 5 (8.88%) | 11 (14.23%) |  | **5.41 (3.08,9.52)** | **3.87 (1.29,11.59)** | **4.04 (2.11,7.73)** |  |
| 0.3< | 8-9 | 93 (32.11%) | 17 (6.71%) | 76 (25.40%) |  | **6.03 (3.98,9.15)** | 1.77 (0.82,3.80) | **5.17 (3.57,7.48)** |  |
| 0.3< | 0-7 | 407 (32.05%) | 149 (10.39%) | 258 (21.66%) |  | **5.74 (3.82,8.62)** | **4.63 (2.55,8.41)** | **3.82 (2.78,5.24)** |  |

Cox regression models were used for all-cause mortality; Fine-Gray models were used for CVD related and non-CVD related mortality.

All percentages are weighted. All models are adjusted for age, education level, and race.

Higher LS7 quartiles indicate better cardiovascular health, lower FI indicate better overall health

Hazard ratio for all-cause mortality is weighted; all mortality rate percentages are weighted. HR = hazard ratio, SHR = sub-distributional hazard ratio, CI = confidence interval, CV = cardiovascular, FI = frailty index, LS7 = Life’s Simple 7 score, * = above 1.00.

##### **T****able S4.** Combined effect of FI and LS7 on all-cause mortality in males across ages.

| **FI Level** | **LS7 Level** | **N** | **Non-CVD mortality**  **N (%)** | **Hazard Ratio (95% CI)** | | | | |
| --- | --- | --- | --- | --- | --- | --- | --- | --- |
|  |  |  |  | Age 30 | Age 40 | Age 50 | Age 60 | Age 70 |
| 0.0-0.1 | 10-14 | 3296 | 127 (2.6%) | Reference | | | | |
| 0.0-0.1 | 8-9 | 4089 | 244 (4.0%) | 1.24 (0.69,2.22) | 1.14 (0.75,1.73) | 1.04 (0.77,1.40) | 0.95 (0.72,1.25) | 0.87 (0.60,1.26) |
| 0.0-0.1 | 0-7 | 4339 | 407 (7.3%) | **2.59 (1.47,4.58)** | **2.07 (1.37,3.14)** | **1.66 (1.23,2.24)** | **1.33 (1.01,1.74)** | 1.06 (0.75,1.51) |
| 0.1-0.2 | 10-14 | 387 | 77 (14.0%) | 1.31 (0.40,4.34) | 1.35 (0.54,3.39) | 1.39 (0.72,2.69) | 1.44 (0.92,2.24) | **1.48 (1.03,2.14)** |
| 0.1-0.2 | 8-9 | 871 | 178 (14.6%) | 0.79 (0.30,2.10) | 0.88 (0.41,1.86) | 0.98 (0.57,1.68) | 1.09 (0.75,1.58) | 1.21 (0.87,1.69) |
| 0.1-0.2 | 0-7 | 1711 | 423 (19.8%) | **3.79 (2.08,6.93)** | **3.14 (1.99,4.97)** | **2.61 (1.86,3.65)** | **2.16 (1.64,2.83)** | **1.79 (1.32,2.42)** |
| 0.2-0.3 | 10-14 | 91 | 29 (20.0%) | 3.77 (0.40,35.34) | 3.09 (0.53,18.04) | 2.53 (0.68,9.35) | 2.07 (0.85,5.03) | 1.69 (0.93,3.07) |
| 0.2-0.3 | 8-9 | 359 | 133 (28.5%) | 1.91 (0.52,6.92) | 1.92 (0.70,5.29) | 1.94 (0.92,4.10) | **1.96 (1.17,3.30)** | **1.98 (1.33,2.97)** |
| 0.2-0.3 | 0-7 | 892 | 298 (28.8%) | **3.48 (1.47,8.24)** | **3.14 (1.65,5.96)** | **2.83 (1.82,4.39)** | **2.55 (1.88,3.44)** | **2.29 (1.67,3.15)** |
| 0.3< | 10-14 | 53 | 26 (47.6%) | **10.01 (1.89,53.03)** | **7.91 (2.06,30.34)** | **6.25 (2.21,17.73)** | **4.94 (2.25,10.86)** | **3.91 (2.07,7.39)** |
| 0.3< | 8-9 | 230 | 103 (35.1%) | 1.73 (0.64,4.67) | 2.01 (0.93,4.36) | **2.33 (1.31,4.16)** | **2.71 (1.75,4.19)** | **3.15 (2.10,4.73)** |
| 0.3< | 0-7 | 794 | 363 (42.6%) | **7.39 (3.80,14.37)** | **6.45 (3.94,10.56)** | **5.63 (3.97,7.99)** | **4.91 (3.71,6.51)** | **4.29 (3.06,6.00)** |

All percentages are weighted. All models are adjusted for age, education level, diagnosis of CVD, NHANES cycle number, and race.

Hazard ratio for all-cause mortality is weighted.

Higher LS7 quartiles indicate better cardiovascular health, lower FI indicate better overall health.

CI = confidence interval, FI = frailty index, LS7 = Life’s Simple 7 score, * = above 1.00.

Bolded text indicate significance at alpha <0.05.

##### **Table S5.** Combined effect of FI and LS7 on CVD mortality in males across ages.

| **FI Level** | **LS7 Level** | **N** | **Non-CVD mortality**  **N (%)** | **Sub-distributional Hazard Ratio (95% CI)** | | | | |
| --- | --- | --- | --- | --- | --- | --- | --- | --- |
|  |  |  |  | Age 30 | Age 40 | Age 50 | Age 60 | Age 70 |
| 0.0-0.1 | 10-14 | 3,296 | 23 (0.5%) | Reference | | | | |
| 0.0-0.1 | 8-9 | 4,089 | 66 (1.1%) | 0.82 (0.29,2.33) | 0.97 (0.43,2.15) | 1.14 (0.63,2.07) | 1.35 (0.83,2.20) | 1.59 (0.93,2.73) |
| 0.0-0.1 | 0-7 | 4,339 | 114 (1.8%) | 1.61 (0.62,4.16) | 1.69 (0.82,3.49) | **1.78 (1.04,3.05)** | **1.87 (1.19,2.95)** | **1.97 (1.17,3.31)** |
| 0.1-0.2 | 10-14 | 387 | 22 (4.4%) | 0.58 (0.07,4.80) | 0.73 (0.14,3.90) | 0.93 (0.26,3.25) | 1.17 (0.48,2.87) | 1.49 (0.75,2.96) |
| 0.1-0.2 | 8-9 | 871 | 59 (4.4%) | 2.15 (0.61,7.58) | 2.10 (0.79,5.58) | 2.05 (0.99,4.25) | **2.00 (1.14,3.50)** | **1.95 (1.13,3.38)** |
| 0.1-0.2 | 0-7 | 1,711 | 159 (6.8%) | **4.89 (1.78,13.41)** | **4.31 (1.98,9.36)** | **3.80 (2.13,6.79)** | **3.35 (2.09,5.37)** | **2.95 (1.78,4.91)** |
| 0.2-0.3 | 10-14 | 91 | 8 (5.2%) | 0.02 (0.00,6.50) | 0.05 (0.00,5.27) | 0.13 (0.00,4.33) | 0.33 (0.03,3.66) | 0.83 (0.20,3.42) |
| 0.2-0.3 | 8-9 | 359 | 39 (7.2%) | 2.02 (0.36,11.24) | 2.06 (0.53,7.99) | 2.10 (0.76,5.83) | **2.14 (1.02,4.52)** | **2.19 (1.18,4.06)** |
| 0.2-0.3 | 0-7 | 892 | 97 (10.2%) | **8.91 (2.90,27.35)** | **6.76 (2.84,16.09)** | **5.13 (2.68,9.81)** | **3.89 (2.33,6.50)** | **2.96 (1.74,5.01)** |
| 0.3< | 10-14 | 53 | 4 (2.6%) | 2.76 (0.11,69.28) | 2.39 (0.18,30.78) | 2.06 (0.30,14.19) | 1.78 (0.44,7.15) | 1.54 (0.52,4.54) |
| 0.3< | 8-9 | 230 | 37 (10.3%) | 1.81 (0.35,9.54) | 2.14 (0.58,7.88) | 2.53 (0.95,6.69) | **2.98 (1.46,6.08)** | **3.52 (1.90,6.50)** |
| 0.3< | 0-7 | 794 | 129 (15.0%) | **14.54 (5.29,39.99)** | **10.78 (4.95,23.51)** | **8.00 (4.45,14.37)** | **5.93 (3.67,9.59)** | **4.40 (2.61,7.40)** |

All percentages are weighted. All models are adjusted for age, education level, diagnosis of CVD, NHANES cycle number, and race.

Higher LS7 quartiles indicate better cardiovascular health, lower FI indicate better overall health

CI = confidence interval, CVD = cardiovascular, FI = frailty index, LS7 = Life’s Simple 7 score

Bolded text indicate significance at alpha <0.05

##### **Table S6.** Association of frailty and cardiovascular health with mortality in females without a CVD diagnosis.

| **Model** | **Term** | **Group** | **N** | | **HR (95% CI)** | | **SHR (95% CI)** | |
| --- | --- | --- | --- | --- | --- | --- | --- | --- |
|  |  |  |  | *All-cause* | | *CVD-related* | |  |
| 1 | 33-FI | Continuous | 16,679 | **1.04 (1.03,1.05)** | | **1.02 (1.01,1.03)** | |  |
|  | LS7 | Continuous | 16,679 | **0.95 (0.91,0.98)** | | **0.89 (0.85,0.94)** | |  |
| 2 | 33-FI | 0.0-0.1 | 10,593 |  | | | |  |
|  |  | 0.1-0.2 | 3,759 | **1.43 (1.23,1.67)** | | **1.41 (1.07,1.87)** | |  |
|  |  | 0.2-0.3 | 1,306 | **2.14 (1.76,2.59)** | | **1.87 (1.37,2.55)** | |  |
|  |  | 0.3< | 1,021 | **3.69 (3.04,4.47)** | | **2.26 (1.65,3.10)** | |  |
|  | LS7 | 3^rd^ tertile | 4,770 |  | | | |  |
|  |  | 2^nd^ tertile | 5,264 | **1.35 (1.05,1.74)** | | 0.89 (0.60,1.34) | |  |
|  |  | 1^st^ tertile | 6,645 | **1.50 (1.19,1.90)** | | **1.53 (1.05,2.21)** | |  |
| 3 | 40-FI | Continuous | 16,679 | **1.05 (1.04,1.05)** | | **1.03 (1.02,1.04)** | |  |
| 4 | 40-FI | 0.0-0.1 | 5,378 |  | | | |  |
|  |  | 0.1-0.2 | 7,288 | **1.51 (1.09,2.08)** | | **1.31 (1.03,1.66)** | |  |
|  |  | 0.2-0.3 | 2,505 | **2.22 (1.60,3.08)** | | **1.85 (1.44,2.39)** | |  |
|  |  | 0.3< | 1,508 | **4.59 (3.23,6.53)** | | **3.40 (2.62,4.41)** | |  |

Cox regression models were used for all-cause mortality; Fine-Gray models were used for CVD-related mortality. All models are adjusted for age, education level, NHANES cycle number, and race. Higher LS7 tertiles indicate better cardiovascular health, lower FI indicate better overall health. Hazard ratio for all-cause mortality is weighted; all mortality rate percentages are weighted. *HR* = hazard ratio, *SHR* = sub-distributional hazard ratio, *CI* = confidence interval, *CVD* = cardiovascular, *FI* = frailty index, *LS7* = Life’s Simple 7 score, 33-FI = FI with 33 items, 40-FI = 33-item FI combined with 7 items from the LS7. Bolded text indicates alpha < 0.05.

##### **Table S7.** Association of frailty and cardiovascular health with mortality in males without a CVD diagnosis at ages 30, 50, and 70.

| **Model** | **Term** | **Group** | **N** | **HR (95% CI)**  *All-cause mortality* | | |  | **SHR (95% CI)**  *CVD-related mortality* | | |
| --- | --- | --- | --- | --- | --- | --- | --- | --- | --- | --- |
|  |  |  |  | ***Age 30*** | ***Age 50*** | ***Age 70*** |  | ***Age 30*** | ***Age 50*** | ***Age 70*** |
| 1 | 33-FI | Continuous | 15,133 | **1.03 (1.01,1.04)** | **1.04 (1.03,1.04)** | **1.04 (1.04,1.05)** |  | **1.02 (1.01,1.04)** | **1.03 (1.02,1.04)** | **1.03 (1.03,1.04)** |
|  | LS7 | Continuous |  | **0.80 (0.73,0.87)** | **0.88 (0.84,0.92)** | 0.97 (0.93,1.00) |  | **0.92 (0.85,0.99)** | **0.95 (0.91,0.99)** | 0.98 (0.95,1.02) |
| 2 | 33-FI | 0.0-0.1 | 11,258 | Reference | | |  | Reference | | |
|  |  | 0.1-0.2 | 2,356 | 1.25 (0.78,2.00) | **1.44 (1.12,1.85)** | **1.67 (1.43,1.95)** |  | 1.21 (0.75,1.94) | 1.27 (0.99,1.64) | **1.34 (1.14,1.56)** |
|  |  | 0.2-0.3 | 913 | 1.33 (0.63,2.84) | **1.71 (1.14,2.57)** | **2.20 (1.83,2.64)** |  | 1.31 (0.66,2.58) | **1.56 (1.07,2.27)** | **1.85 (1.53,2.24)** |
|  |  | 0.3< | 606 | **3.16 (1.77,5.64)** | **3.59 (2.59,4.99)** | **4.09 (3.34,5.01)** |  | **2.49 (1.34,4.61)** | **2.76 (1.96,3.88)** | **3.06 (2.52,3.72)** |
|  | LS7 | 3^rd^ tertile | 3,671 | Reference | | |  | Reference | | |
|  |  | 2^nd^ tertile | 5,064 | 1.03 (0.64,1.68) | 1.01 (0.76,1.35) | 0.99 (0.79,1.25) |  | 1.00 (0.65,1.54) | 0.96 (0.76,1.23) | 0.93 (0.76,1.14) |
|  |  | 1^st^ tertile | 6,398 | **2.46 (1.58,3.84)** | **1.67 (1.29,2.16)** | **1.14 (0.91,1.42)** |  | 1.39 (0.92,2.10) | 1.19 (0.95,1.50) | 1.03 (0.85,1.24) |
| 3 | 40-FI | Continuous | 15,133 | **1.03 (1.02,1.05)** | **1.04 (1.03,1.05)** | **1.05 (1.05,1.05)** |  | **1.03 (1.01,1.05)** | **1.04 (1.02,1.05)** | **1.04 (1.03,1.05)** |
| 4 | 40-FI | 0.0-0.1 | 5,817 | Reference | | |  | Reference | | |
|  |  | 0.1-0.2 | 6,659 | 1.18 (0.76,1.83) | **1.56 (1.22,2.00)** | **2.08 (1.54,2.80)** |  | 1.34 (0.87,2.04) | **1.51 (1.20,1.89)** | **1.70 (1.27,2.28)** |
|  |  | 0.2-0.3 | 1,724 | 1.66 (0.88,3.14) | **2.39 (1.67,3.42)** | **3.43 (2.49,4.73)** |  | 1.46 (0.79,2.70) | **1.89 (1.34,2.65)** | **2.45 (1.80,3.32)** |
|  |  | 0.3< | 933 | **2.94 (1.52,5.72)** | **4.35 (3.02,6.26)** | **6.43 (4.71,8.77)** |  | **2.46 (1.31,4.65)** | **3.22 (2.27,4.58)** | **4.21 (3.08,5.77)** |

Cox regression models were used for all-cause mortality; Fine-Gray models were used for CVD-related mortality. All models are adjusted for age, education level, NHANES cycle number, and race. Higher LS7 tertiles indicate better cardiovascular health, lower FI indicate better overall health. Hazard ratios for all-cause mortality are weighted; all mortality rate percentages are weighted. *HR* = hazard ratio, *SHR* = sub-distributional hazard ratio, *CI* = confidence interval, *CVD* = cardiovascular, *FI* = frailty index, *LS7* = Life’s Simple 7 score, 33-FI = FI with 33 items, 40-FI = 33-item FI combined with 7 items from the LS7. Bolded text indicates alpha < 0.05.

##### **Table S8.** Characteristics of participants excluded due to incomplete cardiovascular information.

|  | **Final Sample** | **Excluded due to missing cardiovascular health data** |
| --- | --- | --- |
| **N** | 35,207 | 9,570 |
| **Age (Mean ± SD)** | 46.6 ± 16.7 | 47.1 ± 18.3 |
| **Sex, female, N (%)** | 18,095 (51.4%) | 5,153 (55.7%) |
| **Race, N (%)** |  |  |
| White | 16,960 (70.8%) | 3,751 (59.1%) |
| Black | 6,846 (10.3%) | 2,392 (14.4%) |
| Hispanic | 9,101 (13.3%) | 2,261 (15.0%) |
| Other | 2,300 (5.6%) | 1,094 (11.5%) |
| **Education, N (%)** |  |  |
| <9th grade | 4,246 (5.9%) | 1,585 (9.9%) |
| 9-11th grade | 5,503 (12.1%) | 1,636 (14.7%) |
| High school | 8,182 (24.0%) | 2,180 (24.3%) |
| Some college associated education | 9,829 (30.8%) | 2,345 (28.1%) |
| College graduate | 7,447 (27.2%) | 1,752 (23.0%) |
| **Frailty Index, N (%)** |  |  |
| <0.1 | 22,538 (70.4%) | 2,245 (61.0%) |
| 0.1-0.2 | 7,084 (17.5%) | 781 (17.1%) |
| 0.2-0.3 | 2,983 (6.8%) | 410 (8.3%) |
| >0.3 | 2,602 (5.3%) | 718 (13.6%) |
| Mean ± SD | 0.09 ± 0.10 | 0.13 ± 0.14 |

All percentages, means, standard deviations are weighted

SD = standard deviation

##### **Table S9.** Mortality rates by frailty and Life’s Simple 7 score groups in males.

|  | **Group** | **N** | **Mortality Rate, N (%)** | | |
| --- | --- | --- | --- | --- | --- |
|  |  |  | *All-cause* | *CVD-related* | |
| **Total Sample** |  | 17,112 | 2,408 (9.3%) | | 757 (2.7%) |
| **33-FI** | 0.0-0.1 | 11,724 | 778 (4.7%) | | 203 (1.2%) |
|  | 0.1-0.2 | 2,969 | 678 (17.3%) | | 240 (5.7%) |
|  | 0.2-0.3 | 1,342 | 460 (28.0%) | | 144 (9.0%) |
|  | 0.3< | 1,077 | 492 (41.3%) | | 170 (13.4%) |
| **LS7** | 3^rd^ tertile | 3,827 | 259 (4.2%) | | 57 (0.9%) |
|  | 2^nd^ tertile | 5,549 | 658 (7.2%) | | 201 (2.0%) |
|  | 1^st^ tertile | 7,736 | 1,491 (14.3%) | | 499 (4.5%) |
| **40-FI** | 0.0-0.1 | 5,901 | 201 (2.4%) | | 37 (0.5%) |
|  | 0.1-0.2 | 7,275 | 849 (8.4%) | | 256 (2.4%) |
|  | 0.2-0.3 | 2,326 | 672 (23.09%) | | 226 (7.1%) |
|  | 0.3< | 1,610 | 686 (38.1%) | | 238 (13.1%) |

All mortality rate percentages are weighted. CVD = cardiovascular, FI = frailty index, LS7 = Life’s Simple 7 score, 33-FI = FI with 33 items, 40-FI = 33-item FI combined with 7 items from the LS7.

##### **Table S10.** Association of frailty and cardiovascular health with non-CVD mortality in females.

| **Model** | **Term** | **Group** | **N** | **Non-CVD Mortality Rate, N (%)** | **SHR (95% CI)** |
| --- | --- | --- | --- | --- | --- |
| 1 | 33-FI | Continuous | 18,095 | 1266 (5.6%) | **1.03 (1.03,1.03)** |
|  | LS7 | Continuous | 18,095 | 1266 (5.6%) | **0.95 (0.93,0.98)** |
| 2 | 33-FI | 0.0-0.1 | 10,814 | 321 (2.3%) | Reference |
|  |  | 0.1-0.2 | 4,115 | 338 (7.2%) | **1.38 (1.18,1.62)** |
|  |  | 0.2-0.3 | 1,641 | 262 (15.3%) | **1.99 (1.67,2.37)** |
|  |  | 0.3< | 1,525 | 345 (22.1%) | **2.95 (2.46,3.53)** |
|  | LS7 | 3^rd^ tertile | 4,860 | 122 (1.7%) | Reference |
|  |  | 2^nd^ tertile | 5,577 | 357 (5.4%) | 1.22 (0.99,1.51) |
|  |  | 1^st^ tertile | 7,658 | 787 (8.9%) | 1.23 (1.00,1.51) |
| 3 | 40-FI | Continuous | 18,095 | 1266 (5.6%) | **1.04 (1.03,1.04)** |
| 4 | 40-FI | 0.0-0.1 | 5,420 | 90 (1.2%) | Reference |
|  |  | 0.1-0.2 | 7,593 | 365 (4.1%) | **1.35 (1.06,1.70)** |
|  |  | 0.2-0.3 | 2,921 | 347 (11.1%) | **1.99 (1.55,2.54)** |
|  |  | 0.3< | 2,161 | 464 (21.1%) | **3.46 (2.69,4.45)** |

Fine-Gray models were used for non-CVD related mortality. All models are adjusted for age, education level, diagnosis of CVD, NHANES cycle number, and race. Higher LS7 tertiles indicate better cardiovascular health, lower FI indicate better overall health. All mortality rate percentages are weighted. SHR = sub-distributional hazard ratio, CI = confidence interval, CVD = cardiovascular, FI = frailty index, LS7 = Life’s Simple 7 score, 33-FI = FI with 33 items, 40-FI = 33-item FI combined with 7 items from the LS7. Bolded text indicates alpha < 0.05.

##### **Table S11.** Associations of frailty and cardiovascular health with non-CVD mortality in males at ages 30, 50, and 70.

| **Model** | **Term** | **Group** | **N** | **Non-CVD Mortality Rate, N (%)** |  | **SHR (95% CI)** | | |
| --- | --- | --- | --- | --- | --- | --- | --- | --- |
|  |  |  |  |  |  | *Age 30* | *Age 50* | *Age 70* |
| 1 | 33-FI | Continuous | 17,112 | 1,651 (6.6%) |  | **1.02 (1.01,1.04)** | **1.03 (1.02,1.03)** | **1.03 (1.03,1.03)** |
|  | LS7 | Continuous | 17,112 | 1,651 (6.6%) |  | **0.91 (0.84,0.98)** | **0.94 (0.90,0.98)** | 0.98 (0.95,1.01) |
| 2 | 33-FI | 0.0-0.1 | 11,724 | 575 (3.6%) |  | Reference | | |
|  |  | 0.1-0.2 | 2,969 | 438 (11.6%) |  | 1.16 (0.74,1.80) | 1.25 (0.98,1.58) | **1.34 (1.17,1.54)** |
|  |  | 0.2-0.3 | 1,342 | 316 (19.0%) |  | 1.42 (0.80,2.51) | **1.65 (1.20,2.27)** | **1.92 (1.64,2.25)** |
|  |  | 0.3< | 1,077 | 322 (27.9%) |  | **2.47 (1.49,4.11)** | **2.63 (1.98,3.49)** | **2.79 (2.37,3.29)** |
|  | LS4 | 3^rd^ tertile | 3,827 | 202 (3.3%) |  | Reference | | |
|  |  | 2^nd^ tertile | 5,549 | 457 (5.2%) |  | 0.98 (0.65,1.49) | 0.92 (0.73,1.17) | 0.86 (0.73,1.03) |
|  |  | 1^st^ tertile | 7,736 | 992 (9.8%) |  | 1.47 (0.99,2.17) | 1.22 (0.98,1.53) | 1.02 (0.86,1.20) |
| 3 | 40-FI | Continuous | 17,112 | 1,651 (6.6%) |  | **1.03 (1.01,1.04)** | **1.03 (1.02,1.04)** | **1.04 (1.03,1.04)** |
| 4 | 40-FI | 0.0-0.1 | 11,724 | 164 (2.0%) |  | Reference | | |
|  |  | 0.1-0.2 | 2,969 | 593 (6.1%) |  | 1.30 (0.86,1.97) | **1.43 (1.14,1.78)** | **1.57 (1.21,2.04)** |
|  |  | 0.2-0.3 | 1,342 | 446 (15.9%) |  | 1.57 (0.90,2.72) | **1.92 (1.42,2.61)** | **2.36 (1.80,3.11)** |
|  |  | 0.3< | 1,077 | 448 (25.0%) |  | **2.53 (1.45,4.39)** | **3.07 (2.26,4.18)** | **3.73 (2.82,4.94)** |

Fine-Gray models were used for non-CVD related mortality. All models are adjusted for age, education level, diagnosis of CVD, NHANES cycle number, and race. Higher LS7 tertiles indicate better cardiovascular health, lower FI indicate better overall health. All mortality rate percentages are weighted. SHR = sub-distributional hazard ratio, CI = confidence interval, CVD = cardiovascular, FI = frailty index, LS7 = Life’s Simple 7 score, 33-FI = FI with 33 items, 40-FI = 33-item FI combined with 7 items from the LS7. Bolded text indicates alpha < 0.05.

##### **Table S12.** Combined effect of FI and LS7 on non-CVD mortality in males across ages.

| **FI Level** | **LS7 Level** | **N** | **Non-CVD mortality**  **N (%)** | **Sub-distribution Hazard Ratio (95% CI)** | | | | |
| --- | --- | --- | --- | --- | --- | --- | --- | --- |
|  |  |  |  | Age 30 | Age 40 | Age 50 | Age 60 | Age 70 |
| 0.0-0.1 | 10-14 | 3296 | 104 (2.1%) | Reference | | | | |
| 0.0-0.1 | 8-9 | 4089 | 178 (2.9%) | 1.26 (0.78,2.05) | 1.11 (0.78,1.59) | 0.98 (0.76,1.28) | 0.87 (0.68,1.10) | 0.77 (0.57,1.04) |
| 0.0-0.1 | 0-7 | 4339 | 293 (5.5%) | **1.68 (1.05,2.69)** | **1.46 (1.04,2.07)** | 1.28 (0.99,1.64) | 1.11 (0.89,1.39) | 0.97 (0.73,1.28) |
| 0.1-0.2 | 10-14 | 387 | 55 (9.7%) | 2.05 (0.72,5.85) | 1.82 (0.81,4.09) | 1.62 (0.90,2.91) | 1.44 (0.95,2.17) | 1.28 (0.90,1.82) |
| 0.1-0.2 | 8-9 | 871 | 119 (10.2%) | 0.59 (0.21,1.62) | 0.67 (0.31,1.48) | 0.77 (0.43,1.36) | 0.88 (0.60,1.30) | 1.00 (0.74,1.36) |
| 0.1-0.2 | 0-7 | 1711 | 264 (13.0%) | **2.32 (1.27,4.22)** | **2.01 (1.28,3.17)** | **1.74 (1.25,2.42)** | **1.51 (1.18,1.94)** | **1.31 (1.00,1.71)** |
| 0.2-0.3 | 10-14 | 91 | 21 (14.9%) | **5.43 (1.31,22.44)** | **4.13 (1.36,12.56)** | **3.14 (1.37,7.19)** | **2.39 (1.30,4.36)** | **1.81 (1.08,3.03)** |
| 0.2-0.3 | 8-9 | 359 | 94 (21.3%) | 1.69 (0.52,5.45) | 1.70 (0.68,4.25) | 1.71 (0.87,3.35) | **1.71 (1.08,2.73)** | **1.72 (1.22,2.42)** |
| 0.2-0.3 | 0-7 | 892 | 201 (18.6%) | 2.09 (1.00,4.38) | **2.01 (1.14,3.54)** | **1.92 (1.27,2.91)** | **1.84 (1.37,2.49)** | **1.77 (1.34,2.34)** |
| 0.3< | 10-14 | 53 | 22 (44.9%) | 4.87 (0.72,32.81) | 4.44 (0.99,19.89) | **4.05 (1.34,12.24)** | **3.69 (1.74,7.84)** | **3.37 (1.99,5.70)** |
| 0.3< | 8-9 | 230 | 66 (24.8%) | **3.95 (1.47,10.62)** | **3.42 (1.58,7.40)** | **2.97 (1.68,5.24)** | **2.57 (1.71,3.87)** | **2.23 (1.57,3.18)** |
| 0.3< | 0-7 | 794 | 234 (27.6%) | **3.59 (1.85,6.94)** | **3.32 (2.01,5.50)** | **3.08 (2.14,4.44)** | **2.86 (2.17,3.76)** | **2.65 (1.99,3.51)** |

All percentages are weighted. All models are adjusted for age, education level, diagnosis of CVD, NHANES cycle number, and race.

Higher LS7 quartiles indicate better cardiovascular health, lower FI indicate better overall health

CI = confidence interval, CVD = cardiovascular, FI = frailty index, LS7 = Life’s Simple 7 score

Bolded text indicate significance at alpha <0.05

##### **Table S13.** Demographic statistics of all males and females by age groups.

|  |  | **Age Groups** | | | | | | |
| --- | --- | --- | --- | --- | --- | --- | --- | --- |
| **LS7 Tertile** | ***Total*** | ***20-29*** | ***30-39*** | ***40-49*** | ***50-59*** | ***60-69*** | ***70-85*** |  |
| **Sample Size, N** | 35,207 | 6,324 | 6,054 | 6,051 | 5,191 | 5,596 | 5,991 |  |
| **Age (Mean ± SD)** | 46.6 ± 16.7 | 24.41 ± 2.90 | 34.59 ± 2.91 | 44.49 ± 2.83 | 54.16 ± 2.85 | 64.10 ± 2.82 | 76.38 ± 4.31 |  |
| **Female, N (%)** | 18,095 (51.4%) | 3,411 (49.49%) | 3,176 (50.32%) | 3,110 (50.85%) | 2,592 (50.74%) | 2,804 (51.94%) | 2,989 (57.57%) |  |
| **CVD, N (%)** | 3,391 (7.57%) | 41 (0.62%) | 89 (1.35%) | 246 (3.69%) | 459 (7.44%) | 928 (16.23%) | 1,626 (27.09%) |  |
| **Race, N (%)** | | | | | | | | |
| White | 16,960 (70.8%) | 2,525 (60.62%) | 2,758 (63.89%) | 2,650 (70.1%) | 2,519 (75.41%) | 2,409 (79.09%) | 4,092 (84.69%) |  |
| Black | 6,846 (10.3%) | 1,293 (12.26%) | 1,134 (11.28%) | 1,254 (10.85%) | 1,088 (9.91%) | 1,285 (8.65%) | 784 (6.59%) |  |
| Hispanic | 9,101 (13.2%) | 1,972 (19.86%) | 1,654 (18.23%) | 1,721 (13.45%) | 1,229 (9.42%) | 1,618 (7.83%) | 901 (5.62%) |  |
| Other | 2,300 (5.6%) | 532 (7.27%) | 505 (6.59%) | 420 (5.6%) | 351 (5.26%) | 277 (4.43%) | 211 (3.1%) |  |
| **Education, N (%)** | | | | | | | | |
| <9th grade | 4,246 (5.9%) | 369 (3.49%) | 429 (4.61%) | 594 (4.78%) | 583 (5.12%) | 1,014 (7.82%) | 1,252 (13.17%) |  |
| 9-11th grade | 5,503 (12.1%) | 1,098 (13.16%) | 923 (12.28%) | 911 (10.86%) | 759 (10.76%) | 870 (11.23%) | 937 (15.01%) |  |
| High school | 8,182 (24.0%) | 1,538 (24.11%) | 1,355 (22%) | 1,355 (23.66%) | 1,142 (22.7%) | 1,278 (25.15%) | 1,507 (28.12%) |  |
| Some college | 9,829 (30.8%) | 2,250 (38.45%) | 1,721 (29.84%) | 1,738 (30.24%) | 1,489 (31.15%) | 1,361 (27.71%) | 1,269 (24.24%) |  |
| College graduate | 7,447 (27.2%) | 1,067 (20.8%) | 1,623 (31.27%) | 1,447 (30.45%) | 1,214 (30.27%) | 1,066 (28.08%) | 1,023 (19.46%) |  |
| **Mortality Rate, N (%)** | | | | | | | | |
| All-cause | 4,228 (8.5%) | 118 (1.54%) | 125 (1.76%) | 270 (3.54%) | 375 (6.37%) | 850 (13.41%) | 2,485 (37.93%) |  |
| CVD related | 1,311 (2.4%) | 107 (1.44%) | 106 (1.51%) | 212 (2.75%) | 281 (4.89%) | 564 (8.97%) | 1,645 (25.91%) |  |
| Non-CVD related | 2,917 (6.1%) | 11 (0.1%) | 19 (0.25%) | 58 (0.8%) | 94 (1.48%) | 286 (4.44%) | 840 (12.02%) |  |
| **Number of Prescription Medications, N (%)** | | | | | | | | |
| 8+ | 27,662 (80.82%) | 6,192 (97.39%) | 5,709 (93.85%) | 5,260 (86.27%) | 3,945 (76.52%) | 3,525 (61.66%) | 3,027 (49.49%) |  |
| 4-7 | 5,711 (14.79%) | 115 (2.37%) | 294 (5.34%) | 621 (11.33%) | 923 (17.72%) | 1,561 (28.98%) | 2,197 (37.26%) |  |
| 0-3 | 1,813 (4.39%) | 15 (0.24%) | 48 (0.81%) | 164 (2.4%) | 319 (5.76%) | 503 (9.36%) | 764 (13.25%) |  |
| **33-Item Frailty Index, N (%)** | | | | | | | | |
| <0.1 | 22,538 (70.4%) | 5,563 (90.44%) | 5,037 (85.6%) | 4,555 (77.44%) | 3,328 (68.39%) | 2,507 (48.95%) | 1,536 (26.2%) |  |
| 0.1-0.2 | 7,084 (17.5%) | 656 (8.09%) | 746 (10.59%) | 914 (14.13%) | 1,004 (18.6%) | 1,667 (29.67%) | 2,091 (36.02%) |  |
| 0.2-0.3 | 2,983 (6.8%) | 75 (1.06%) | 180 (2.59%) | 286 (4.53%) | 397 (6.37%) | 771 (12.67%) | 1,271 (21.19%) |  |
| >0.3 | 2,602 (5.3%) | 28 (0.41%) | 88 (1.22%) | 290 (3.9%) | 458 (6.64%) | 644 (8.7%) | 1,090 (16.6%) |  |
| Mean ± SD | 0.09 ± 0.10 | 0.04 ± 0.05 | 0.05 ± 0.06 | 0.08 ± 0.09 | 0.10 ± 0.10 | 0.13 ± 0.11 | 0.19 ± 0.12 |  |

All percentages, means, standard deviations are weighted.

CVD = cardiovascular disease.

##### **Table S14.** Cardiovascular health behaviors and factors by age groups.

|  |  | **Age Groups** | | | | | | |
| --- | --- | --- | --- | --- | --- | --- | --- | --- |
| **Age Groups** | ***Total*** | ***20-29*** | ***30-39*** | ***40-49*** | ***50-59*** | ***60-69*** | ***70-85*** |  |
| **Sample Size**, *N* | 35,207 | 6,324 | 6,324 | 6,324 | 6,324 | 6,324 | 6,324 |  |
| **Smoking Status**, *N (%)* | | | | | | | | |
| Never or quit > 12 months prior | 18,761 (52.8%) | 3,947 (59.15%) | 3,560 (56.8%) | 3,255 (53.33%) | 2,486 (48.05%) | 2,537 (44.62%) | 2,965 (50.65%) |  |
| Former, quit ≤ 12 months prior | 8,920 (25.0%) | 713 (11.86%) | 929 (17.04%) | 1,170 (21.32%) | 1,452 (30.23%) | 2,068 (39.68%) | 2,577 (42.35%) |  |
| Current Smoker | 7,526 (22.2%) | 1,662 (29%) | 1,562 (26.16%) | 1,620 (25.35%) | 1,249 (21.72%) | 984 (15.7%) | 446 (7%) |  |
| **Body Mass Index** (kg/m^2^), *N (%)* | | | | | | | | |
| <25.0 | 10,535 (31.9%) | 2,731 (45.49%) | 1,862 (33.42%) | 1,573 (28.19%) | 1,255 (26.17%) | 1,265 (23.83%) | 1,839 (31.37%) |  |
| 25.0-29.9 | 12,165 (34.1%) | 1,812 (28.55%) | 2,013 (32.88%) | 2,147 (35.75%) | 1,813 (35.37%) | 2,008 (35.78%) | 2,366 (38.76%) |  |
| ≥30.0 | 12,507 (34.0%) | 1,779 (25.96%) | 2,176 (33.7%) | 2,325 (36.06%) | 2,119 (38.46%) | 2,316 (40.38%) | 1,783 (29.88%) |  |
| Mean ± SD | 27.16 ± 0.48 | 27.0 ± 6.6 | 28.5 ± 6.7 | 29.0 ± 6.6 | 29.3 ± 6.5 | 29.4 ± 6.2 | 27.9 ± 5.5 |  |
| **Physical Activity** (minutes/week), *N (%)* | | | | | | | | |
| 150 moderate or vigorous activity | 15,634 (49.5%) | 3,458 (59.37%) | 3,050 (53.45%) | 2,891 (52.17%) | 2,258 (47.92%) | 2,131 (42.66%) | 1,837 (32.16%) |  |
| 1-149 moderate or vigorous activity | 8,804 (25.0%) | 1,581 (22.96%) | 1,612 (27.24%) | 1,534 (25.55%) | 1,313 (25.27%) | 1,384 (24.3%) | 1,376 (23.61%) |  |
| No activity | 10,769 (25.5%) | 1,283 (17.67%) | 1,389 (19.31%) | 1,620 (22.28%) | 1,616 (26.81%) | 2,074 (33.04%) | 2,775 (44.23%) |  |
| Mean ± SD | 318.13 ± 34.85 | 427.7 ± 565.0 | 356.3 ± 528.8 | 343.4 ± 493.7 | 319.8 ± 524.8 | 273.0 ± 482.0 | 200.8 ± 399.7 |  |
| **Healthy Diet Score†**, *N (%)* | | | | | | | | |
| 4-5 goals met | 520 (1.2%) | 44 (0.56%) | 55 (0.83%) | 65 (0.81%) | 95 (1.52%) | 120 (1.93%) | 141 (2.39%) |  |
| 2-3 goals met | 12,696 (34.7%) | 1,752 (26.98%) | 1,921 (31.32%) | 2,074 (32.73%) | 2,071 (39.55%) | 2,343 (41.29%) | 2,524 (41.52%) |  |
| 0-1 goals met | 21,991 (64.1%) | 4,526 (72.45%) | 4,075 (67.85%) | 3,906 (66.45%) | 3,021 (58.93%) | 3,126 (56.78%) | 3,323 (56.09%) |  |
| **Total Cholesterol** (mg/dL), *N (%)* | | | | | | | | |
| <200 untreated | 15,965 (46.1%) | 4,484 (74.15%) | 3,449 (57.69%) | 2,749 (44.18%) | 1,691 (30.27%) | 1,661 (27.43%) | 1,925 (29.36%) |  |
| 200-239/treated to goal | 13,946 (39.1%) | 1,274 (19.23%) | 1,826 (29.97%) | 2,304 (38.89%) | 2,455 (49.08%) | 2,896 (54.28%) | 3,178 (55.07%) |  |
| ≥240 | 5,296 (14.8%) | 564 (6.63%) | 776 (12.34%) | 992 (16.93%) | 1,041 (20.65%) | 1,032 (18.29%) | 885 (15.57%) |  |
| Mean ± SD | 197.7 ± 41.7 | 180.0 ± 37.1 | 195.1 ± 39.2 | 204.3 ± 40.4 | 208.5 ± 42.2 | 202.8 ± 42.3 | 196.5 ± 44.0 |  |
| **Fasting Blood Glucose** (mg/dL), *N (%)* | | | | | | | | |
| <100 untreated | 5,701 (90.38%) | 4,966 (83.57%) | 4,404 (75.78%) | 3,170 (65.49%) | 2,887 (56.08%) | 2,974 (51.82%) | 5,701 (90.38%) |  |
| 100-125 or treated to goal | 511 (7.95%) | 855 (13.16%) | 1,173 (18.22%) | 1,333 (24.47%) | 1,712 (29.73%) | 2,052 (33.43%) | 511 (7.95%) |  |
| ≥126 | 110 (1.68%) | 230 (3.27%) | 468 (5.99%) | 684 (10.04%) | 990 (14.19%) | 962 (14.75%) | 110 (1.68%) |  |
| Mean ± SD | 97.5 ± 31.9 | 87.6 ± 17.5 | 91.8 ± 25.9 | 96.5 ± 31.2 | 102.5 ± 38.5 | 106.5 ± 38.6 | 107.0 ± 34.0 |  |
| **Blood Pressure** (mmHg), *N (%)* | | | | | | | | |
| SBP<120 or DBP<80 untreated. | 4,471 (69.13%) | 3,634 (59.54%) | 2,534 (42.81%) | 1,380 (28.07%) | 830 (17.1%) | 557 (10.09%) | 4,471 (69.13%) |  |
| SBP 120-139 \| DBP 80-89 \| treated to goal. | 1,659 (27.61%) | 1,970 (33.62%) | 2,656 (44.68%) | 2,705 (52.7%) | 2,917 (53.68%) | 2,824 (47.65%) | 1,659 (27.61%) |  |
| SBP ≥140 or DBP ≥90 | 192 (3.26%) | 447 (6.84%) | 855 (12.5%) | 1,102 (19.23%) | 1,842 (29.22%) | 2,607 (42.27%) | 192 (3.26%) |  |
| Mean SBP ± SD | 122.2 ± 17.7 | 113.6 ± 11.2 | 115.5 ± 12.7 | 119.6 ± 14.5 | 124.9 ± 16.9 | 131.0 ± 19.0 | 138.1 ± 22.1 |  |
| Mean DBP ± SD | 70.8 ± 12.4 | 66.5 ± 11.2 | 71.5 ± 10.8 | 74.7 ± 10.6 | 74.7 ± 10.9 | 71.0 ± 12.8 | 63.9 ± 16.0 |  |

All percentages, means, standard deviations are weighted.

†The five Healthy Diet Score goals are: (1) fruits and vegetables ≥ 4.5 cups/day, (2) fish ≥ two 3.5-ounce servings/week, (3) whole grains ≥ three 1 oz. servings/day, (4) sodium < 1500 mg/day, and (5) added sugar in sugar-sweetened beverages < 450 kcal/week.

DBP = diastolic blood pressure, SBP = systolic blood pressure, SD = standard deviation.

## Supplementary Figures

##### **Figure S1.** Proportion of participants in each Life’s Simple 7 score tertile by frailty index level (33-item version) for males and females. 3rd, 2nd, and 1st tertiles represent Life’s Simple 7 scores (LS7) 10-14, 8-9, 0-7, respectively. All percentages are weighted. Higher Life’s Simple 7 scores indicate better cardiovascular health and lower frailty index scores indicate better overall health.

##### **Figure S2.** Simple slopes of the association between Life’s Simple 7 score and the 33-item frailty index from a linear regression model for males and females. Shaded ribbons represent the 95% confidence intervals. Higher Life’s Simple 7 score indicate better cardiovascular health and lower frailty index indicates better overall health.

**
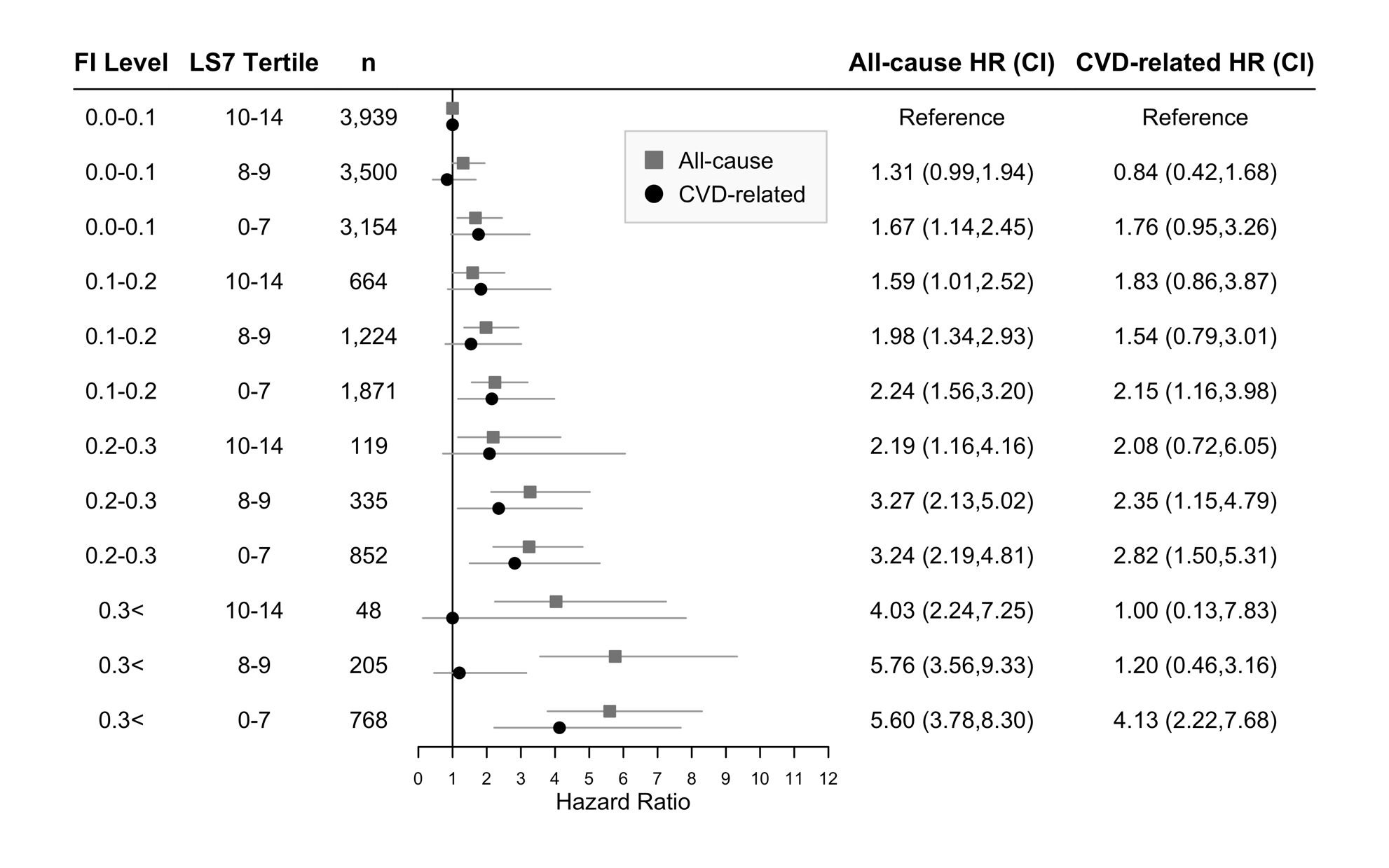
**

##### **Figure S3.** Cox regression and Fine-Gray models for combined effect of Life’s Simple 7 score and frailty on all-cause and CVD-related mortality in females without a CVD diagnosis. All models were adjusted for age, education level, NHANES cycle number, and race. The 95% confidence interval is indicated by grey lines. FI = frailty index, LS7 = Life’s Simple 7 score, CVD = cardiovascular disease, CI = confidence interval, n = number of participants, * = p > 0.05. LS7 for 3^rd^, 2^nd^, and 1^st^ tertiles are 10-14, 8-9, and 0-7, respectively. The 0.0-0.1 FI and 3^rd^ LS7 tertile represent the healthiest group, while the 0.3< FI and 1st LS7 tertile represent the least healthy group; all other groups are intermediary between these two extremes. The 33-item FI was used in this forest plot.

**
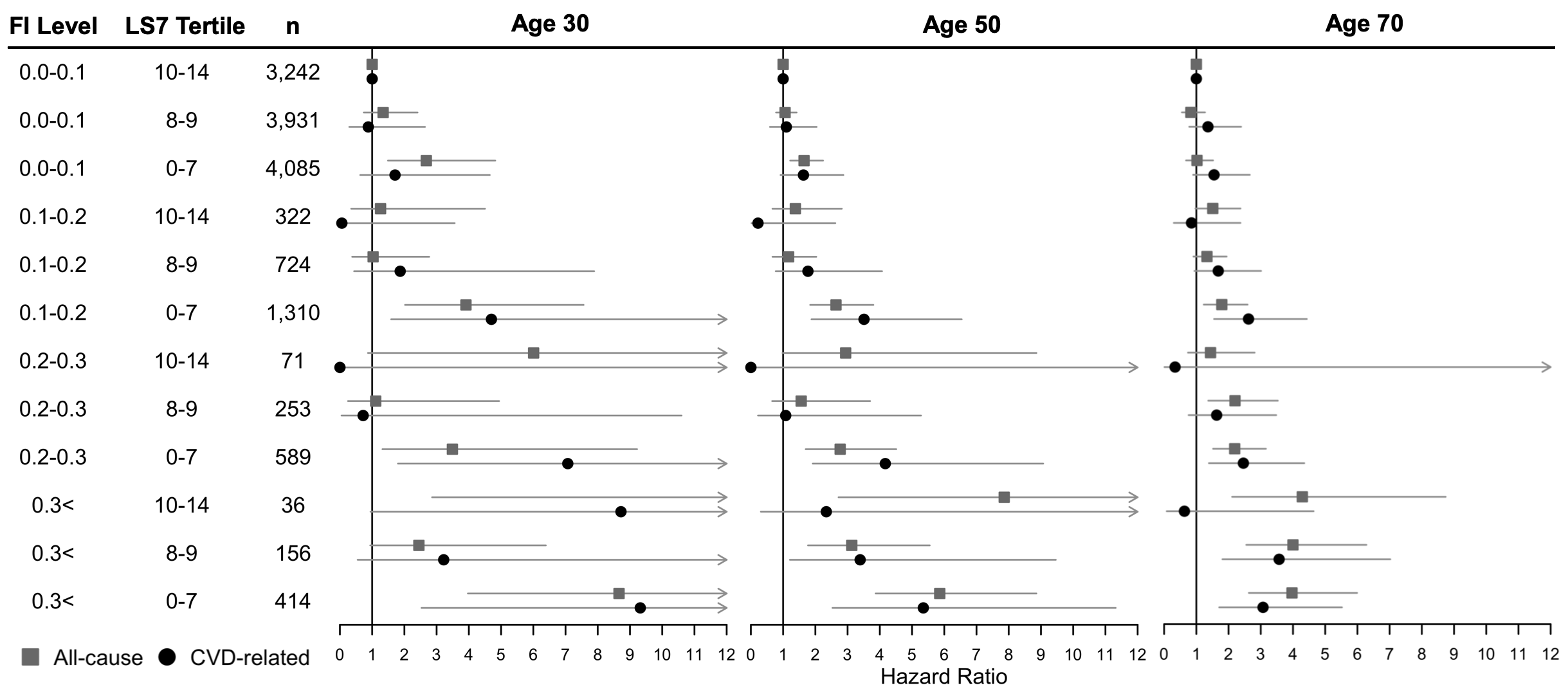
**

##### **Figure S4.** Cox regression and Fine-Gray models for combined effect of Life’s Simple 7 score and frailty on all-cause and CVD-related mortality in males without a CVD diagnosis, with age centered at 30, 50, and 70. All models were adjusted for age, education level, NHANES cycle number, and race. The 95% confidence interval is indicated by grey lines. FI = frailty index, LS7 = Life’s Simple 7 score, CVD = cardiovascular disease, CI = confidence interval, n = number of participants, * = p > 0.05. LS7 for 3rd, 2nd, and 1st tertiles are 10-14, 8-9, and 0-7, respectively. The 0.0-0.1 FI and 3rd LS7 tertile represent the healthiest group (reference group), while the 0.3< FI and 1st LS7 tertile represent the least healthy group; all other groups are intermediary between these two extremes. The 33-item FI was used in this forest plot.


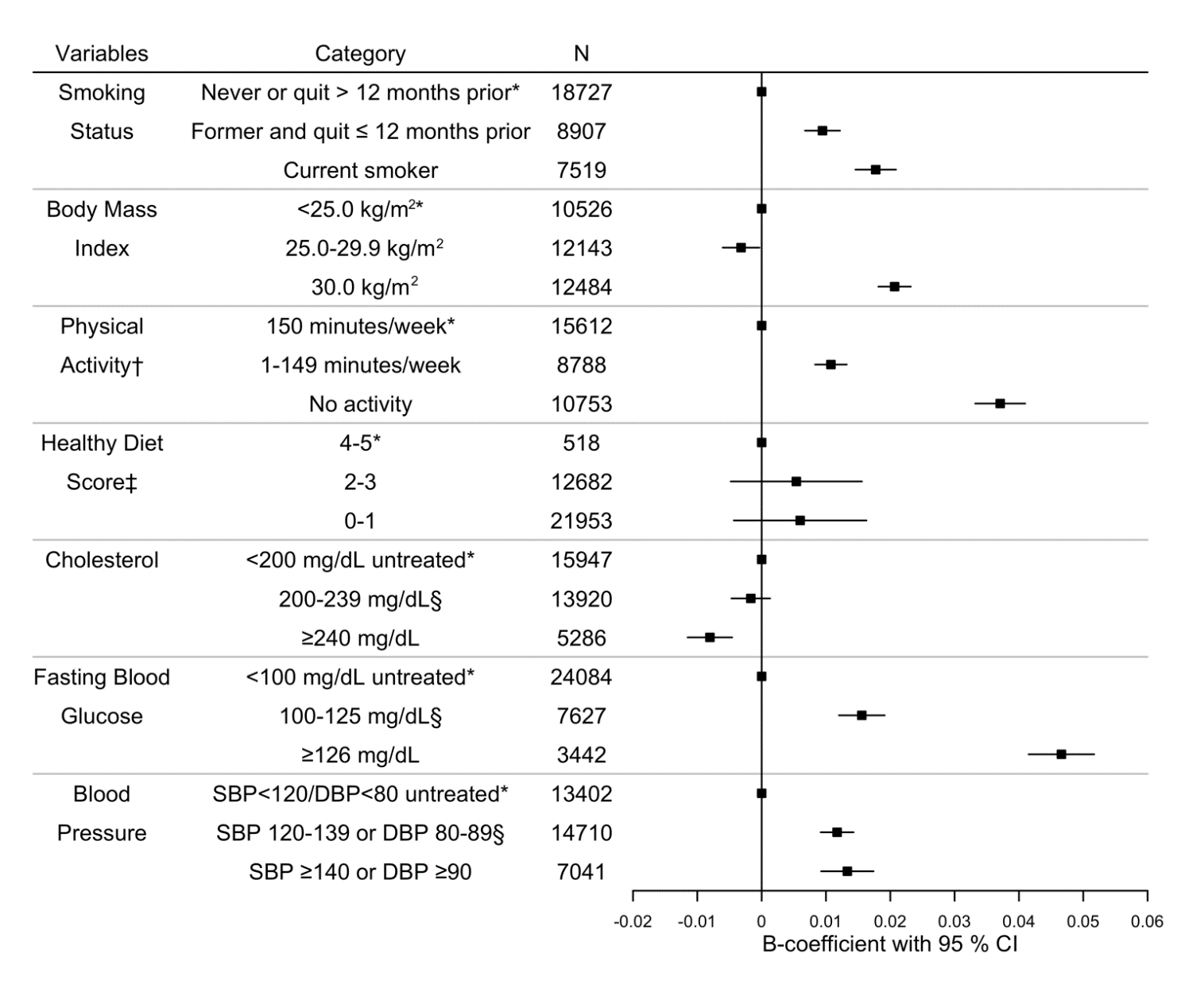


##### **Figure S5.** Multiple linear regression model for the association between individual cardiovascular health metrics and frailty. All models are weighted and adjusted for age, sex, education level, and race. *Reference group, †Physical activity minutes are for both moderate and vigorous activity, ‡The five Healthy Diet Score goals are: (1) fruits and vegetables ≥ 4.5 cups/day, (2) fish ≥ two 3.5-ounce servings/week, (3) whole grains ≥ three 1 oz. servings/day, (4) sodium < 1500 mg/day, and (5) added sugar in sugar-sweetened beverages < 450 kcal/week, §Within the range or treated to goal. CI = confidence interval, N = number of participants.
